# Differential impact of transplantation on peripheral and tissue-associated viral reservoirs: Implications for HIV gene therapy

**DOI:** 10.1371/journal.ppat.1006956

**Published:** 2018-04-19

**Authors:** Christopher W. Peterson, Jianbin Wang, Claire Deleage, Sowmya Reddy, Jasbir Kaur, Patricia Polacino, Andreas Reik, Meei-Li Huang, Keith R. Jerome, Shiu-Lok Hu, Michael C. Holmes, Jacob D. Estes, Hans-Peter Kiem

**Affiliations:** 1 Clinical Research Division, Fred Hutchinson Cancer Research Center, Seattle, WA, United States of America; 2 Department of Medicine, University of Washington, Seattle, WA, United States of America; 3 Sangamo Therapeutics, Richmond, CA, United States of America; 4 AIDS and Cancer Virus Program, Frederick National Laboratory for Cancer Research, Leidos Biomedical Research, Inc., Frederick, MD, United States of America; 5 Washington National Primate Research Center, Seattle, WA, United States of America; 6 Vaccine and Infectious Diseases Division, Fred Hutchinson Cancer Research Center, Seattle, WA, United States of America; 7 Department of Laboratory Medicine, University of Washington, Seattle, WA, United States of America; 8 Department of Pharmaceutics, University of Washington, Seattle, WA, United States of America; 9 Department of Pathology, University of Washington, Seattle, WA, United States of America; Emory University, UNITED STATES

## Abstract

Autologous transplantation and engraftment of HIV-resistant cells in sufficient numbers should recapitulate the functional cure of the Berlin Patient, with applicability to a greater number of infected individuals and with a superior safety profile. A robust preclinical model of suppressed HIV infection is critical in order to test such gene therapy-based cure strategies, both alone and in combination with other cure strategies. Here, we present a nonhuman primate (NHP) model of latent infection using simian/human immunodeficiency virus (SHIV) and combination antiretroviral therapy (cART) in pigtail macaques. We demonstrate that transplantation of CCR5 gene-edited hematopoietic stem/progenitor cells (HSPCs) persist in infected and suppressed animals, and that protected cells expand through virus-dependent positive selection. CCR5 gene-edited cells are readily detectable in tissues, namely those closely associated with viral reservoirs such as lymph nodes and gastrointestinal tract. Following autologous transplantation, tissue-associated SHIV DNA and RNA levels in suppressed animals are significantly reduced (p ≤ 0.05), relative to suppressed, untransplanted control animals. In contrast, the size of the peripheral reservoir, measured by QVOA, is variably impacted by transplantation. Our studies demonstrate that CCR5 gene editing is equally feasible in infected and uninfected animals, that edited cells persist, traffic to, and engraft in tissue reservoirs, and that this approach significantly reduces secondary lymphoid tissue viral reservoir size. Our robust NHP model of HIV gene therapy and viral persistence can be immediately applied to the investigation of combinatorial approaches that incorporate anti-HIV gene therapy, immune modulators, therapeutic vaccination, and latency reversing agents.

## Author summary

Over the past decade, multiple strategies have been investigated for HIV Cure. Especially notable are cell-based approaches, inspired by the cure of the Berlin Patient, who was transplanted with hematopoietic stem cells from a donor that carried a mutation at the CCR5 locus. This mutation renders cells resistant to infection with most strains of HIV. Our goal in this study was to apply a safer version of this curative approach to more patients, using gene editing to generate a similar CCR5 mutation in a patient’s own cells. In a nonhuman primate model, we show that hematopoietic stem cells from infected, antiretroviral therapy-suppressed animals can be isolated, gene edited, and transplanted back into the infected host. Following transplantation, gene edited cells give rise to progeny, namely T-cells, that are protected against infection and hence gain a selective advantage. Most importantly, we show that these cells traffic to and reduce the size of "viral reservoirs" in secondary tissue sites that contribute to the persistence of HIV, for example in patients on antiretroviral therapy.

## Introduction

Timothy Brown, known as the Berlin Patient, has recently reached 11 years of HIV-free remission in the absence of combination antiretroviral therapy (cART) [1–3]. Intensive studies have postulated three central tenants that led to his functional cure. First, the conditioning regimen that was administered prior to transplantation with allogeneic hematopoietic stem and progenitor cells (HSPCs) helped to clear the primary hematological malignancy, and also facilitated engraftment of donor HSPCs [4, 5]. Although conditioning also likely ablated a proportion of latently infected cells, we have demonstrated that the corresponding loss of virus-specific immunity offsets this benefit [6]. In addition, the ability of conditioning regimens such as myeloablative total body irradiation (TBI) to target viral reservoirs in tissues is limited [7]. Hence, the conditioning regimen plays an important role in gene therapy-mediated cure of HIV infection, but is most likely insufficient to induce cART-independent virological remission.

The second tenant of remission/cure in the Berlin patient was the infusion of allogeneic donor cells, and the resultant “graft versus HIV” effect. Despite close HLA matching between host and donor products, donor cells still frequently recognize host cells as foreign, and destroy these cells through well-characterized immunological mechanisms [5, 8]. Graft-vs.-tumor effects are an essential component of effective allogeneic stem cell transplantation strategies for various leukemias [9, 10], contributing to reduction of tumor burden and engraftment of donor stem cells. Therefore, pathologies associated with graft-versus-host disease must be closely regulated in transplant patients to balance safety and efficacy [11, 12]. In the setting of latent HIV infection, allogeneic donor cells are likely to target reservoir cells for destruction, although the frequency of targeting of infected versus uninfected host cells has not been characterized. Two HIV^+^ Boston Patients received allogeneic HSPC products and experienced substantial periods of virus-free remission, but did eventually rebound [13–15]. Collectively, these clinical cases suggest that the graft-versus-HIV effect contributed to the Berlin Patient’s cure, but was likely insufficient for virus eradication.

The third and arguably most important tenant of remission/cure in the Berlin patient was gene-specific HIV resistance, conferred by the homozygous CCR5Δ32 mutant allogeneic donor cells (not present in the Boston patients’ donor cells). CCR5Δ32 is well characterized in regard to HIV resistance [16, 17], and in other pathologies [18–20], yet is not associated with significantly impaired quality of life. Notably, the Berlin patient ended cART concurrent with his first HSPC transplant with no subsequent evidence of virus recrudescence [3]. In contrast, our recent findings suggest that the early days and weeks following conditioning and transplantation provide an ideal environment for viral replication, especially in the absence of effective cART [6, 21]. Together, these data strongly suggest that virus-protected donor cells played a crucial and immediate role in the Berlin Patient’s functional cure, and should be considered an essential facet of any cure strategy.

CCR5Δ32 donor cells are rare, compounded by the difficulties in identifying an HLA-matched CCR5Δ32 donor. Furthermore, the toxicities of allogeneic transplantation and myeloablative conditioning prevent broad applicability to otherwise healthy, well-suppressed HIV^+^ patients. In contrast, autologous transplantation is safer and applicable to more patients [22]. We have previously demonstrated that autologous transplantation with CCR5 gene-edited HSPCs is safe and feasible, and results in long-term engraftment of CCR5-edited HSPC progeny [23]. Here, we conducted transplants with ΔCCR5 HSPCs in a robust nonhuman primate (NHP) model of suppressed HIV infection. Our goals were i) to evaluate the feasibility of biallelic ΔCCR5 gene therapy in animals infected with simian/human immunodeficiency virus (SHIV) relative to our previous data in uninfected animals, ii) compare the kinetics of ΔCCR5 cell engraftment in the presence or absence of unsuppressed viral replication, and iii) to evaluate the ability of our *in vivo* model of HIV latency to recapitulate key features of viral reservoirs in patients.

## Materials and methods

### Ethics statement

This study was carried out in strict accordance with the recommendations in the *Guide for the Care and Use of Laboratory Animals of the National Institutes of Health* (”The Guide”), and was approved by the Institutional Animal Care and Use Committees of the Fred Hutchinson Cancer Research Center and University of Washington, Protocol # 3235–03. All animals were housed at and included in standard monitoring procedures prescribed by the Washington National Primate Research Center (WaNPRC). This included at least twice-daily observation by animal technicians for basic husbandry parameters (e.g., food intake, activity, stool consistency, overall appearance) as well as daily observation by a veterinary technician and/or veterinarian. Animals were housed in cages approved by “The Guide” and in accordance with Animal Welfare Act regulations. Animals were fed twice daily, and were fasted for up to 14 hours prior to sedation. Environmental enrichment included grouping in compound, large activity, or run-through connected cages, perches, toys, food treats, and foraging activities. If a clinical abnormality was noted, standard WaNPRC procedures were followed to notify the veterinary staff for evaluation and determination for admission as a clinical case. Animals were sedated by administration of Ketamine HCl and/or Telazol and supportive agents prior to all procedures. Following sedation, animals were monitored according to WaNPRC standard protocols. WaNPRC surgical support staff are trained and experienced in the administration of anesthetics and have monitoring equipment available to assist: heart rate, respiration, and blood oxygenation monitoring, audible alarms and LCD readouts, monitoring of blood pressure, temperature, etc. For minor procedures, the presence or absence of deep pain was tested by the toe-pinch reflex. The absence of response (leg flexion) to this test indicates adequate anesthesia for this procedure. Similar parameters were used in cases of general anesthesia, including the loss of palpebral reflexes (eye blink). Analgesics were provided as prescribed by the Clinical Veterinary staff for at least 48 hours after the procedures, and could be extended at the discretion of the clinical veterinarian, based on clinical signs. Decisions to euthanize animals were made in close consultation with veterinary staff, and were performed in accordance with guidelines as established by the American Veterinary Medical Association Panel on Euthanasia (2013). Prior to euthanasia, animals were first rendered unconscious by administration of ketamine HCl.

### SHIV challenge, CCR5 gene editing and autologous transplantation

**Fig 1:**
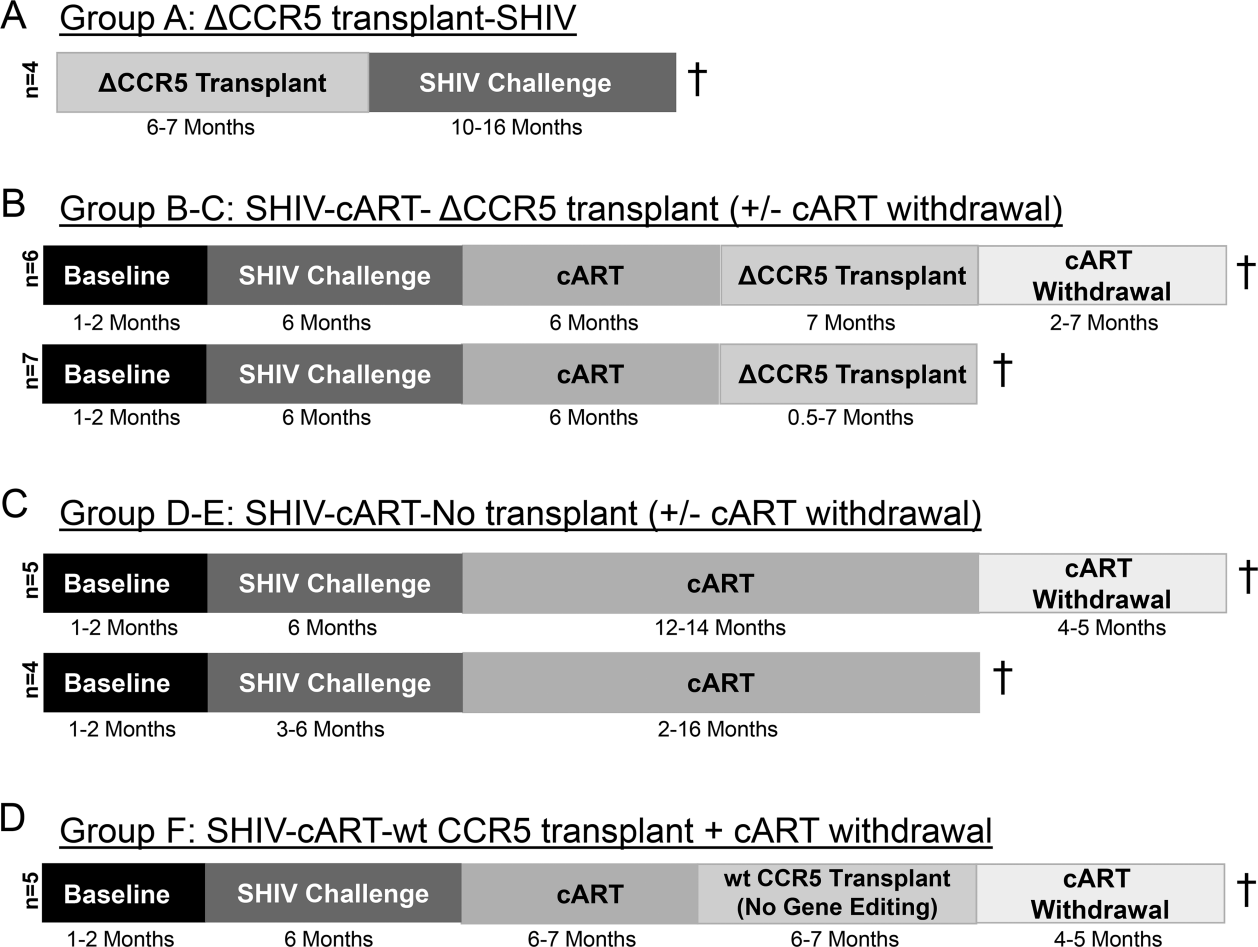
Cohorts for ΔCCR5 transplant study. A total of 31 SHIV-infected pigtail macaques were utilized in this study. **(A)**: Four animals were transplanted with autologous, CCR5 gene edited HSPCs, and recovered for 6–7 months prior to SHIV challenge (“Group A”). **(B)**: Thirteen animals were transplanted following SHIV infection and stable suppression by cART, using an identical protocol to animals in panel A. Six of these animals (“Group B”) underwent cART withdrawal prior to necropsy, and 7 animals (“Group C”) were necropsied while stably suppressed. **(C)**: Two untransplanted control cohorts were also studied. The first contained 5 animals (“Group D”) that were necropsied following infection, suppression, and subsequent cART withdrawal; the second contained 4 animals (“Group E”) that were infected, suppressed, and necropsied following stable suppression of plasma viremia. **(D)**: A third control cohort of 5 animals (“Group F”) was infected, suppressed, and underwent transplant with autologous HSPCs that were not gene edited (“wt CCR5 transplant”) before withdrawal of cART. In Groups B-F, all animals were maintained on cART throughout the transplantation procedure. Daggers (†) indicate necropsy.

**Fig 2:**
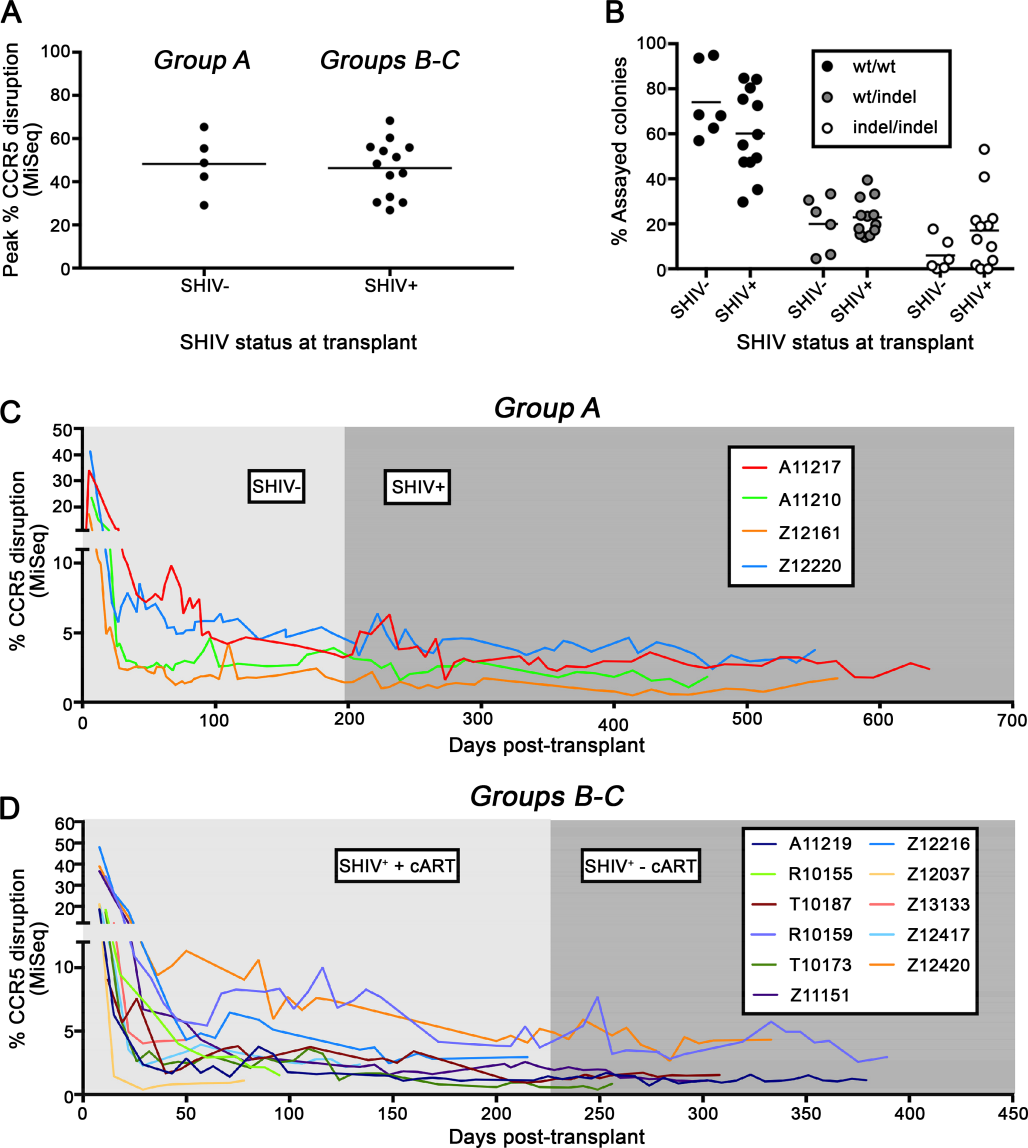
ΔCCR5 cells persist in peripheral blood of SHIV^+^ animals. Following isolation of autologous HSPC from SHIV-naïve (Group A) and SHIV-infected animals (Groups B-C) and *ex vivo* CCR5-gene editing, deep sequencing was used to quantify the percentage of edited CCR5 alleles in the HSPC infusion product and in transplanted animals. **(A):** Peak CCR5 disruption in CD34^+^ HSPC infusion products cultured for 5–12 days *ex vivo*. **(B)**: Colony assay quantifying the percentage of wt, mono-, and bi-allelically disrupted, CD34^+^ HSPC-derived colonies. Error bars represent standard error of the mean for the percentages of at least 48 colonies derived from 5 SHIV- and 12 SHIV+ animals. **(C)**: CCR5 gene editing in peripheral blood samples from previously SHIV-naïve animals before and after SHIV infection. **(D)**: CCR5 gene editing in peripheral blood samples from previously SHIV-infected and cART-suppressed animals before and after release of cART.

Detailed study schematics are shown in **Fig 1** and **S1 Table**. Delivery of CCR5 ZFN mRNA to nonhuman primate HSPCs, and pre-SHIV data for ΔCCR5 Transplant-SHIV animals (Group A) have been previously described [23]. These animals were infected with SHIV-1157ipd3N4 (“SHIV-C”) [24] approximately 200 days following transplantation. SHIV-cART-ΔCCR5 Transplant (Groups B-C) animals were infected identically, without prior transplantation, and suppressed by cART (Tenofovir and FTC kindly provided by Gilead Sciences; Raltegravir kindly provided by Merck) 6 months after infection. Following approximately 6 months of cART, these animals underwent nearly identical transplants relative to the ΔCCR5 Transplant-SHIV cohort. One minor difference between gene editing procedures for Group A and Groups B-C was that CCR5 ZFN mRNA for Groups B-C was resuspended in electroporation buffer rather than water, which may have contributed to slightly increased efficiencies of biallelic editing (**Fig 2B**). Animals in Groups D-E and Group F served as untransplanted and unedited controls for Groups B-C, respectively, and have been previously described [6]. Groups B-F were further used as controls for Group A animals, using data collected prior to initiation of cART (**Fig 1**). All transplanted animals were mobilized with Granulocyte Colony-Stimulating Factor and Stem Cell Factor for 4 days prior to enrichment of bone marrow-derived CD34^+^ HSPCs, received a conditioning regimen consisting of 1020 cGy total body irradiation (TBI), and were subsequently infused with gene-edited, autologous HSPCs as previously described [6, 23]. All SHIV-infected animals were maintained on cART throughout the transplantation procedure. Due to the intensive nature of these experiments, animals were studied in a staggered format, rather than contemporaneously.

### Gene disruption assays

Total genomic DNA was isolated from peripheral blood and tissues at longitudinal and necropsy time points as described previously [6, 21]. The percentage of CCR5-edited alleles in each sample was quantified using the Illumina MiSeq platform [23].

### Plasma viral load assay, tissue preparations, and SHIV PCR

Plasma viral load was measured as described previously [25, 26]. Single tissue pieces <10 mm^3^ were collected and stored overnight in nucleic acid preservative at 4°C, blotted dry, and stored at -80°C prior to homogenization with a Precellys 24 homogenizer and CK28-R hard tissue homogenizing beads (Bertin Corp, Washington, DC). Extraction of total genomic DNA and RNA and quantitation of SHIV copies per genome equivalent and normalized RNA copies were performed as described previously [6, 21]. Briefly, SHIV DNA was normalized to a genomic DNA standard, macaque RNase P p30 subunit (MRPP30), and SHIV RNA was normalized to the cycle threshold (Ct) of MRPP30 RNA. The 95% limit of detection for the assay is 6 copies/reaction; for tissue specimens, the absolute limit of detection varies with the number of input cells.

### Lymph node collections, gut biopsies, and flow cytometry

At the indicated longitudinal time points, lymph nodes were collected from peripheral sites (axillary, inguinal) and flash frozen. Total genomic DNA and RNA were prepared from these tissue samples as described above. Gastrointestinal biopsies from upper GI (duodenum/jejunum) and lower GI (colon) were collected as described [24], and dissociated in RPMI media containing 0.5 mg/mL collagenase and 1 U/mL DNase I. Viability of single cell suspensions was measured using a Guava Cytometer (Merck Millipore, Billerica, MA), and a small aliquot was stained with antibodies including CD3-Ax700 clone SP34-2, CD4-PerCP-Cy5.5 clone L200, CD8-APC-Cy7 clone SK1, CD28-PE-Cy5 clone CD28.2, CD45RA-FITC clone 5H9, CD95-APC clone DX2, CCR7-PE-Cy7 clone 3D12, and CCR5-PE clone 3A9, all from Becton Dickinson (Franklin Lakes, NJ). Total genomic DNA was extracted from the remainder of the sample for SHIV RNA and/or DNA analyses. When nucleic acid yields from these samples were insufficient, flash-frozen GI biopsy pinches collected at the same points were utilized.

### Peripheral blood subset sorting

Peripheral blood sorts from the indicated subsets were performed using magnetic bead kits from Miltenyi Biotec (Bergisch Gladbach, Germany) or via antibody labeling and a FACS ARIA cell sorter (Becton Dickinson). In Group A animals, large-volume peripheral blood draws were collected immediately prior to SHIV infection, and approximately 100 days after SHIV infection. In Group B, draws were collected from infected, suppressed, transplanted animals immediately prior to cART withdrawal, and approximately 100 days after viral rebound following withdrawal of cART. Total genomic DNA was isolated from each bead-sorted sample, as well as from hemolysed total peripheral and bone marrow white blood cells (WBC, BM-WBC), Ficoll-sorted PBMC, and the granulocyte-enriched Ficoll pellet fraction (“GRANS”). Purity of bead-enriched fractions was confirmed by flow cytometry [23]. The percentage of CCR5-edited alleles in each sample was measured using Illumina MiSeq. To calculate SHIV-dependent enrichment in each subset, values during productive SHIV infection (primary infection or post-cART withdrawal viral rebound) were divided by values from pre-infection or cART-suppressed infection time points, respectively.

### Next-generation RNAscope and DNAscope in situ hybridization and quantitative image analysis

Viral RNA (RNAscope) and DNA (DNAscope) detection and quantitative image analysis was performed as previously described [27].

### Quantitative viral outgrowth assay

QVOA assays were performed essentially as described previously [6].

### Statistics

Statistical analyses were performed using unpaired t-tests or nonparametric Mann-Whitney tests and GraphPad Prism 7 software, without assumption of consistent standard deviations between data sets. Throughout the text, error bars represent standard error of the mean (SEM), and p-values are expressed as exact values to 3 decimal places.

## Results

### Study design

Thirty-one pigtail macaques in 6 groups (A-F) of 4–7 animals each were analyzed in this study (**Fig 1**). Prior to SHIV challenge, Group A “ΔCCR5 Transplant-SHIV” animals received CCR5-edited HSPCs that were produced using our previously described CCR5 Zinc Finger Nuclease (ZFN) platform (**Fig 1A**); pre-SHIV data from these animals has been previously described [23]. Groups B and C “SHIV-cART-ΔCCR5 Transplant” received CCR5-edited cells after SHIV-1157ipd3N4 infection and stable suppression by cART, and were necropsied either following cART withdrawal (Group B) or while stably suppressed (Group C) (**Fig 1B**). Three control groups of infected and suppressed animals were utilized. Group D-E animals did not undergo ΔCCR5 transplantation, and were necropsied either following cART withdrawal (Group D) or while stably suppressed (Group E). Group F animals were transplanted with unedited (“wt CCR5”) HSPCs (**Fig 1C**) [6]. Because animals in Groups B-E did not undergo an experimental intervention until they were stably suppressed, data from primary infection served as controls for Group A (animals transplanted prior to infection). A complete list of the animals used in this study can be found in **S1 Table**.

### CCR5-edited HSPCs persist in peripheral blood of SHIV^+^ animals

We have previously shown that CCR5-edited macaque HSPCs engraft in uninfected animals [23]. To model the impact of our approach in cART-suppressed HIV^+^ patients, we measured the engraftment of CCR5-edited HSPCs in cART-suppressed SHIV^+^ animals. We found that the efficiency of CCR5 editing was almost identical in CD34^+^ HSPCs isolated from SHIV^-^ and SHIV^+^ animals (**Fig 2A**). Colony-forming assays showed that HSPCs from SHIV^+^ animals had slightly higher rates of biallelic disruption of CCR5, likely due to incremental improvements in the handling of edited cells *ex vivo* (**Fig 2B**). Following infusion into autologous hosts that had received myeloablative TBI, we observed similar kinetics of engraftment of edited cells in suppressed SHIV^+^ animals, relative to SHIV^-^ controls (**Fig 2C and 2D**). This included a high level of gene editing proportional to that in the respective animals’ HSPC infusion products (**Fig 2A**) at early time points post-transplant, and stable engraftment of edited cells at 3–4% of total peripheral blood at further time points, up to 13 months post-transplant. This data strongly suggests that CCR5 gene editing is equally feasible in infected and uninfected animals, and that edited cells persist comparably in infected and uninfected recipients, providing support for the feasibility of this approach in HIV-infected individuals.

### Lymphoid tissue sites of virus persistence exhibit higher levels of CCR5-edited HSPCs

**Fig 3:**
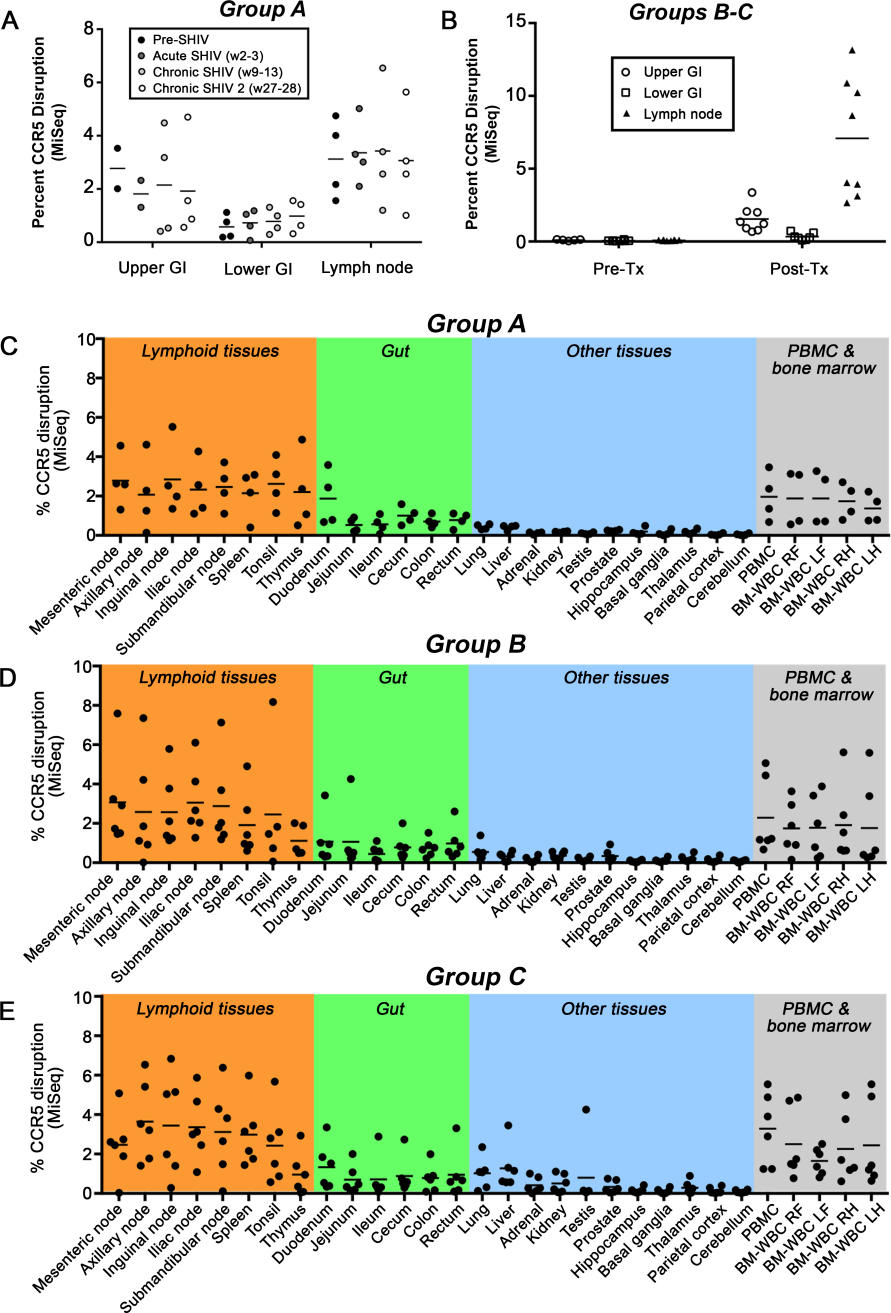
ΔCCR5 cells persist in tissues of SHIV^+^ animals. Tissues were collected longitudinally (figure panels **[A-B]**) or at necropsy (panels **[C-E]**) from transplanted animals, and the percentage of CCR5-edited alleles was quantified from total tissue homogenate by Illumina MiSeq. **(A)** Duodenum/Jejunum (Upper GI), colon (Lower GI) and peripheral lymph nodes from Group A animals transplanted prior to SHIV challenge. **(B)** Same as panel A, from Group B-C animals transplanted following SHIV infection and stable suppression by cART. **(C)** Necropsy tissues from Group A animals as in panel A. **(D)** Necropsy tissues from Group B animals in panel B that were necropsied following cART withdrawal. **(E)** Necropsy tissues from Group C animals in panel B that were necropsied while stably suppressed on cART.

We next evaluated the kinetics of ΔCCR5 cell engraftment in tissues, particularly those implicated in HIV latency, including lymph nodes and gastrointestinal (GI) tract. Notably, tissue measurements did not exclude cells of non-HSPC origin, and therefore likely underestimated the true extent of HSPC-derived ΔCCR5 cell engraftment. In Group A animals that were transplanted first and then SHIV-infected, ΔCCR5 cells made up ~1% (Lower GI) and 1–6% (Upper GI and Lymph Nodes) of the total cells assayed from each tissue (**Fig 3A**). In Group B-C animals that were infected and suppressed prior to transplantation, we observed similar levels of engraftment, with the exception of lymph nodes, in which ΔCCR5 engraftment was greater than 10% in several animals (**Fig 3B**). We next performed necropsies, collected total genomic DNA from 25 tissue sites, and again measured the percentage of CCR5-edited alleles by deep sequencing. In Group A animals that were transplanted with ΔCCR5 cells and then infected with SHIV, we saw the highest level of ΔCCR5 engraftment in lymph nodes, tonsil, and thymus (**Fig 3C**). We observed similar trends in animals that were infected, suppressed, and transplanted with ΔCCR5 HSPCs, necropsied either before (Group B) (**Fig 3D**) or after (Group C) (**Fig 3E**) cART withdrawal and viral rebound. These findings support the notion that ΔCCR5 cells traffic to and persist at lymphoid tissue sites of virus persistence.

### Trends toward delayed viral rebound in CCR5-edited animals

**Fig 4:**
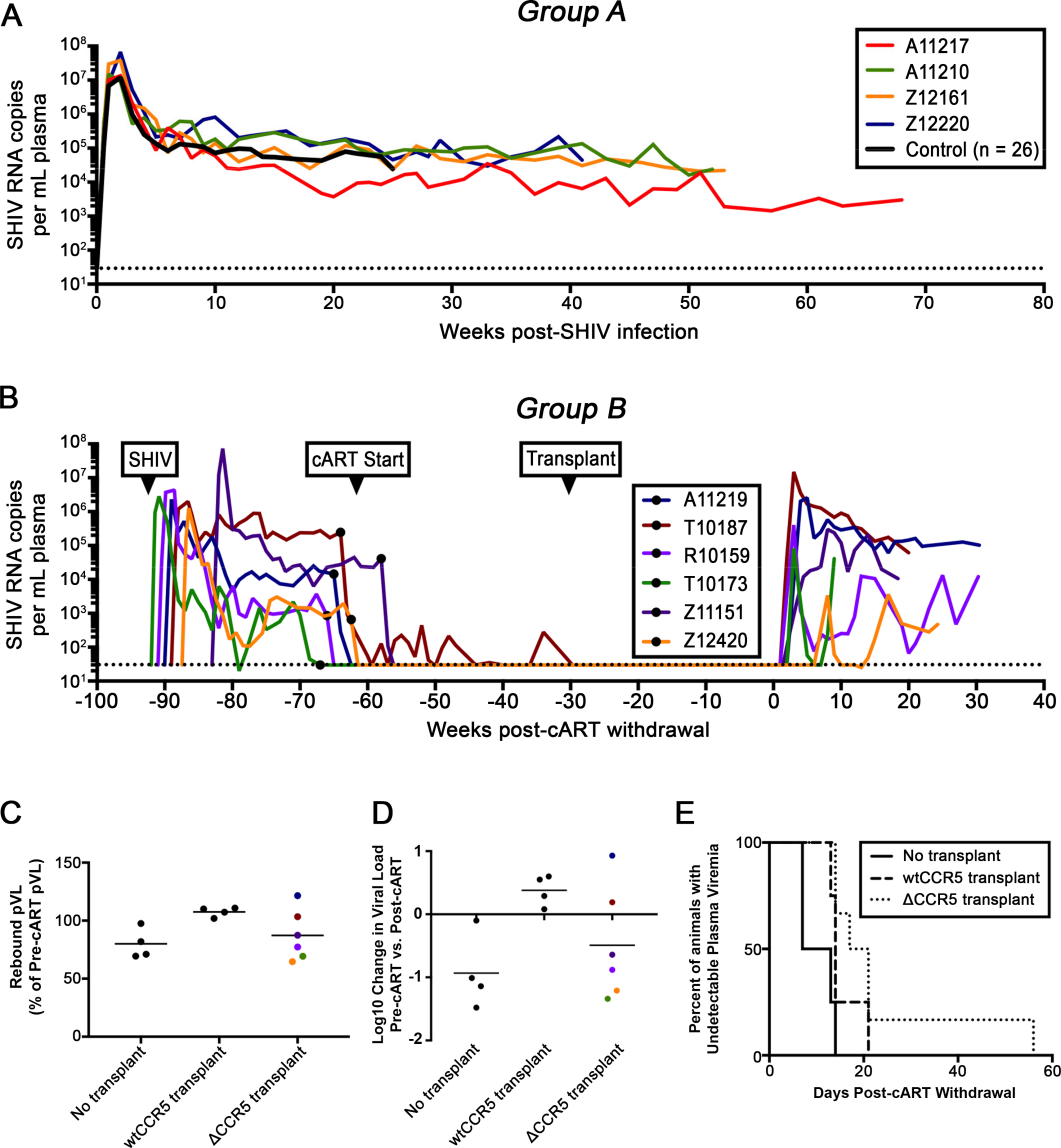
Plasma viral loads in ΔCCR5 animals are comparable to controls. **(A)** SHIV plasma viral loads from Group A animals transplanted prior to infection. **(B)** SHIV plasma viral loads from Group B animals that were transplanted following infection and stable suppression and subsequently released from cART. Black circles indicate cART initiation. **(C)** Measurements from Group B in panel B (“ΔCCR5 Transplant”) were used to calculate the ratio of rebound viremia to viremia during primary infection, and compared to Group C (“No Transplant”) and Group F (“wtCCR5 Transplant”). **(D)** Log change in average set point viral load between rebound and primary infection. **(E)** Days to rebound following cART withdrawal. Colored data points in panels **(C-D)** correspond to lines in **(B)**. Dotted lines in panels **(A-B)** indicate limit of detection of the plasma viral load assay (30 copies/mL).

Our previous findings suggest that a threshold level of infection-resistant HSPCs is capable of improving virus-specific immune responses following SHIV infection [28, 29]. To quantify the impact of ~4% ΔCCR5 cells on suppressed and/or unsuppressed SHIV viremia, we measured viral RNA and DNA in peripheral blood of our animals. First, we compared SHIV plasma viral loads in Group A animals that were transplanted prior to infection to primary infection data from Group B-F animals that were not transplanted prior to infection. We observed no difference between these groups (**Fig 4A**). Plasma viral loads for Groups C and E are shown in **S1** and **S2 Figs**, respectively, while plasma viral loads for Groups D and F have been described previously [6]. Next, we compared the magnitude and kinetics of viral rebound following cART withdrawal in three groups of infected, suppressed animals: Group B (transplanted with ΔCCR5 cells), Group D (untransplanted), and Group F (transplanted with non-modified (“wt CCR5”) cells) (**Fig 4B**). We have previously observed that the magnitude of plasma viral rebound in Group F animals was higher than in Group D [6]. Interestingly, the magnitude of viral rebound in Group B ΔCCR5-transplanted animals was comparable to Group D untransplanted animals (**Fig 4D**). Similarly, the net change in average set point viral load during rebound viremia, relative to primary infection was negative across untransplanted (Group D) and ΔCCR5 transplanted animals (Group B), but positive across wt CCR5 controls (Group F) (**Fig 4D**). Finally, the time to viral rebound trended later in ΔCCR5 transplanted animals, relative to untransplanted and wt CCR5 transplanted controls. Notably, ΔCCR5 animal IDs R10159 and Z12420 did not establish consistent set point viral load following cART withdrawal; plasma viremia in ID Z12420 was not observed for almost 2 months post-cART withdrawal (**Fig 4B–4E**). Although these results did not reach statistical significance, the observed trends suggest that ΔCCR5 transplantation may have a beneficial impact on the time to viral rebound following cART treatment interruption, even at low levels of ΔCCR5 cells.

### Gut-associated central memory CD4^+^ T-cells are preserved in ΔCCR5 animals

**Fig 5:**
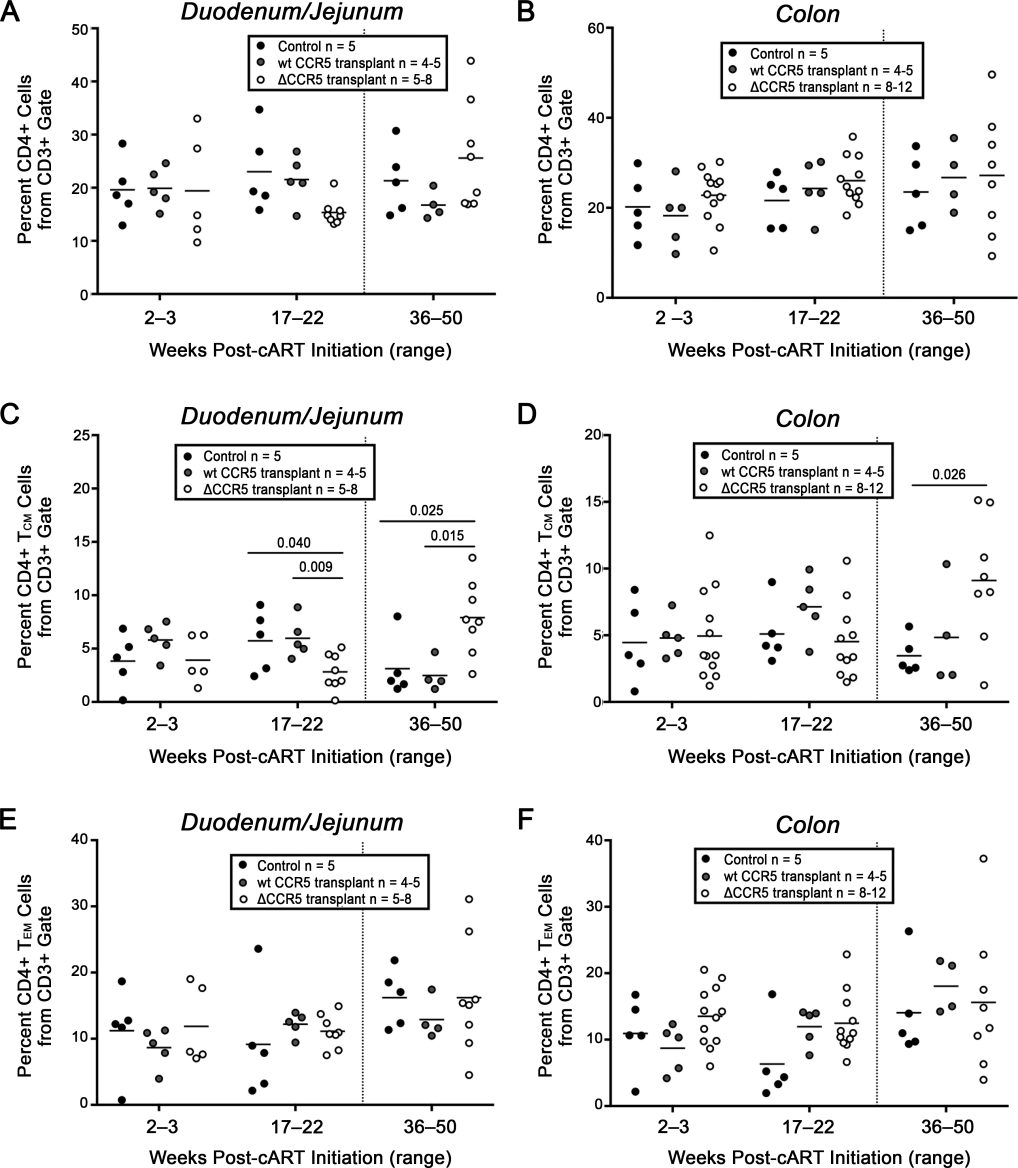
CD4^+^ T-Cell subset percentages in SHIV^+^, cART suppressed animals before and after CCR5 gene editing transplantation. Upper (duodenum/jejunum; panels **A, C, E**) and lower GI biopsies (colon; panels **B, D, F**) were collected from infected and suppressed animals in Groups B-C (“ΔCCR5 Transplant,” open circles), and compared to untransplanted animals in Groups D-E (“Control,” closed circles), and Group F animals that were transplanted with non-edited HSPCs (“wt CCR5 Transplant,” gray circles). Shown are total CD3^+^CD4^+^ cells (panels **A-B**), Central Memory CD4^+^ T-cells (T_CM_, panels **C-D**), and Effector memory CD4^+^ T-cells (T_EM_, panels **E-F**) measured by flow cytometry from enzymatically dissociated specimens. Memory subsets were distinguished on the basis of CD45RA and CCR7 expression (see materials and methods). Dotted line separates pre-transplant and post-transplant time points. Exact p-values are shown where applicable.

In Group A animals that were transplanted with ΔCCR5 cells prior to SHIV infection, longitudinal gastrointestinal (GI) biopsy collections showed comparable levels of SHIV DNA and SHIV RNA relative to untransplanted controls, up to 28 weeks post-infection; similar trends were observed in peripheral lymph node samples (**S3 Fig**). CD4^+^ T-cell percentages from Group A biopsy samples were at or below those of untransplanted controls before and after SHIV infection, likely due to residual immune suppression from the myeloablative conditioning regimen [21] (**S4 Fig**). In Group B-C animals that were ΔCCR5 transplanted following infection and stable suppression, we observed a significant decrease in lymph node-associated SHIV DNA after transplantation and prior to cART withdrawal (36–50 weeks post-cART initiation) relative to a time point immediately prior to transplantation (17–22 weeks post-cART initiation) (p = 0.0004) (**S5 Fig**). However, control samples were not available to contextualize these results as transplantation- vs. ΔCCR5-dependent. Intriguingly, despite an unexplained decrease in gut-associated CD4^+^ central memory cell percentages (CD4^+^ T_CM_) prior to ΔCCR5 transplantation, we observed a significant increase in this subset following ΔCCR5 transplantation, relative to wt CCR5 transplant (Group F) (p = 0.015 in upper GI) and untransplanted controls (Groups D-E) (p = 0.025 in upper GI, and p = 0.026 in lower GI) (**Fig 5**). These findings are consistent with a model in which HSPC-derived ΔCCR5 CD4^+^ T-cells preferentially refill the virus- and TBI-depleted niche in the gut of SHIV^+^ suppressed animals.

### ΔCCR5 memory T-cells undergo virus-dependent positive selection

**Fig 6:**
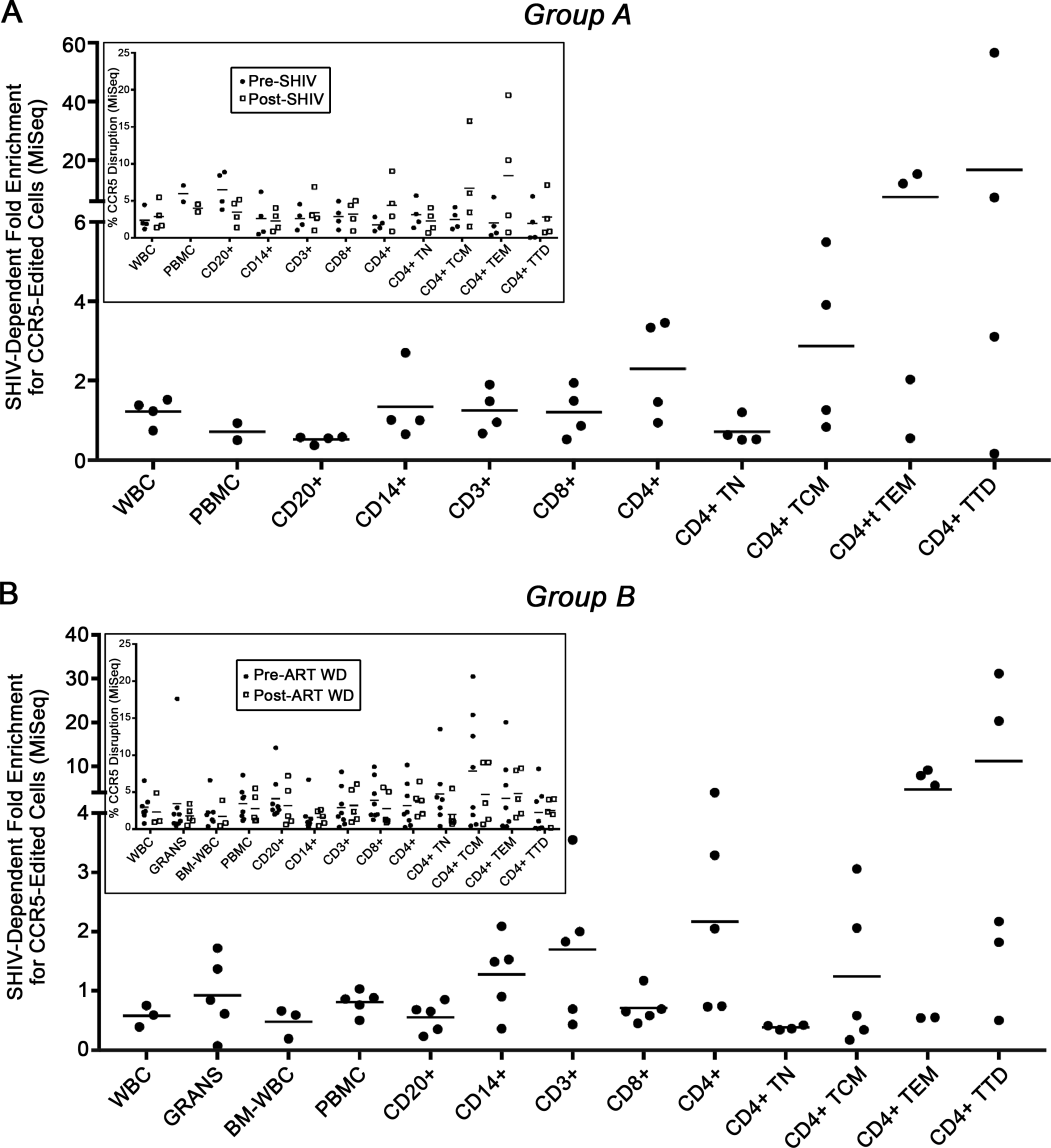
Multi-lineage ΔCCR5 cells undergo subset-specific, virus-dependent positive selection. In Group A animals that were transplanted prior to infection (**A**) or Group B animals that were transplanted following infection and stable suppression by cART (**B**), large volume peripheral blood draws were collected, and PBMC subsets were isolated using magnetic bead- and flow-assisted cell sorting. **(A)** Ratio of CCR5-edited cells post-infection relative to pre-infection in Group A animals. **(B)** Same calculation as (A), except dividing post-cART withdrawal values by pre-cART withdrawal values. Insets: raw values used to calculate ratios. TN: Naïve T-cell; TCM: Central Memory T-cell; TEM: Effector Memory T-cell; TTD: Terminally Differentiated T-Cell.

We observed an increased percentage of ΔCCR5 cells in tissues known to contribute to HIV/SHIV persistence (**Fig 3C–3E**), and found that gut-associated CD4^+^ T_CM_ recovered more rapidly in SHIV^+^, cART-suppressed ΔCCR5 relative to controls (**Fig 5C and 5D**). These data suggest that ΔCCR5 cells might undergo virus-dependent positive selection at these sites. To directly measure preferential expansion of ΔCCR5 cells, we isolated CD4^+^ T-cells from whole blood before and after productive SHIV replication. Our metric for virus-dependent selection was calculated by dividing the proportion of ΔCCR5 CD4^+^ T-cells in each animal during productive infection by the same value measured prior to productive infection. In animals that were transplanted prior to infection (Group A), values measured ~100 days post-SHIV challenge were divided by values measured pre-SHIV challenge (**Fig 6A inset**). In animals that were transplanted following infection and stable suppression (Group B), values measured ~100 days post-cART withdrawal and viral rebound were divided by values measured pre-cART withdrawal (**Fig 6B inset**). In both groups, we observed a ratio <2 (indicating <2-fold virus-dependent selection) in all subsets, with the exception of CD4^+^ T-cells. In CD4^+^ T-cell subsets sorted on the basis of CD45RA and CCR7 expression, a marked increase in virus-dependent positive selection was observed as CD4^+^ T-cells transitioned from naïve to central memory, effector memory, and terminally differentiated phenotypes. In animals that were transplanted prior to infection, enrichments of up to 5-, 15-, and 56-fold were observed in central memory, effector memory, and terminally differentiated subsets, respectively (**Fig 6A**). In animals that were transplanted following infection and stable suppression, enrichments of up to 3-, 9-, and 31-fold were observed in central memory, effector memory, and terminally differentiated subsets, respectively (**Fig 6B**). These data indicate that HSPC-derived ΔCCR5 CD4^+^ T-cells persist *in vivo*, and display trends consistent with resistance to infection with CCR5-tropic SHIV, and virus-dependent positive selection.

### Impact of ΔCCR5 HSPC transplantation on the peripheral SHIV reservoir

**Fig 7:**
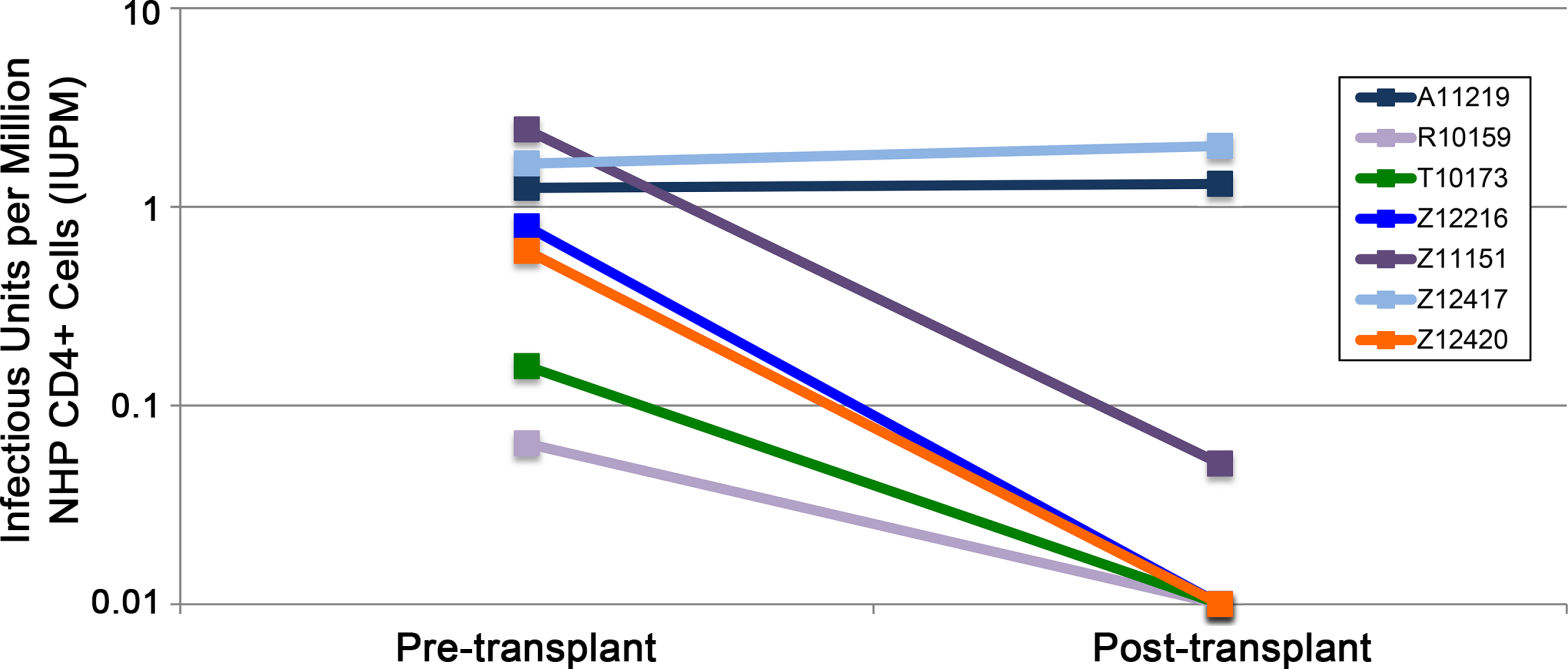
Impact of myeloablative conditioning and ΔCCR5 HSPCs on the size of the peripheral SHIV reservoir. Quantitative viral outgrowth assay (QVOA) was used to measure the size of the latent SHIV reservoir in Group B-C animals (n = 7) that were sampled before and after ΔCCR5 transplantation. An arbitrary value of 0.01 was used for animals in which the viral reservoir was undetectable. “Undetectable” is defined as no p27 ELISA-positive signal, despite seeding of replicate wells with up to 5x10^6^ input CD4^+^ T-cells.

We have previously developed a SHIV-adapted Quantitative Viral Outgrowth Assay (QVOA) to measure the size of the peripheral viral reservoir before and after transplantation in wt CCR5 transplant animals [6]. We found that autologous transplantation with wt CCR5 HSPCs did not significantly impact the size of the peripheral SHIV reservoir, although reservoir size decreased to undetectable levels in 2 out of 4 animals tested. Here, we asked whether ΔCCR5 transplantation impacted peripheral reservoir size. We observed a similar binary trend in ΔCCR5 transplant animals as we previously observed in wt CCR5 transplant animals. Out of 7 SHIV-infected, cART-suppressed animals that were transplanted with ΔCCR5 HSPCs, the measurable inducible reservoir size decreased to undetectable levels in 4 animals, decreased by only 1.5 logs in one animal, and was unchanged in two others (**Fig 7**). As such, the proportion of long-term persisting ΔCCR5 progeny that we were able to achieve in this study were insufficient to significantly impact the size of the latent peripheral SHIV reservoir.

### Tissue-associated SHIV DNA is reduced in ΔCCR5 animals

**Fig 8:**
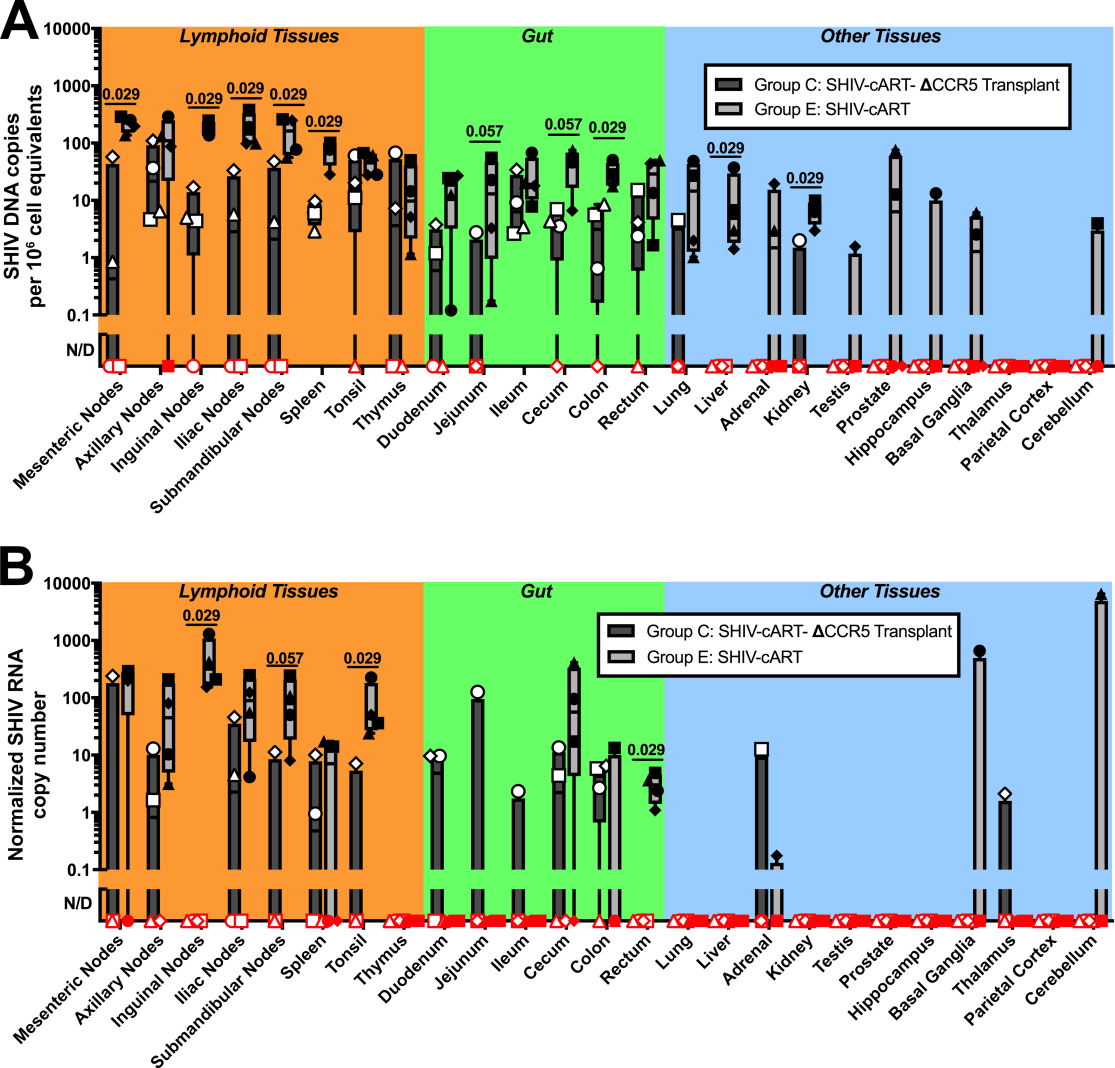
ΔCCR5 transplant decreases tissue viral reservoirs in stably suppressed animals. Following SHIV infection and cART suppression, necropsy and extensive tissue collections were performed on Group E animals (n = 4) that were not transplanted (“SHIV-cART,” light gray bars + closed circles) and Group C animals (n = 4) that were transplanted with CCR5 gene-edited cells (“SHIV-cART-ΔCCR5 Transplant,” dark gray bars + open shapes). All animals were stably suppressed at the time of necropsy, when tissues were collected from the indicated sites, and nucleic acids were extracted from total tissue homogenates. **(A)** SHIV DNA copies per 10^6^ cell equivalents. **(B)** Normalized SHIV RNA copy number. Quantitative PCR was used to measure SHIV DNA and RNA, and normalized to macaque RNase P p30 (MRPP30) DNA and RNA, respectively. Red shapes indicate samples in which SHIV DNA or RNA was not detected (ND) despite 4 (DNA) or 2 (RNA) re-tests of the original negative sample. Positive values represent the quantity on initial testing, or for samples undergoing repeat testing, the average of all results. Cell input for each assay as determined by MRPP30 is shown in S2 Table. Shapes indicate individual values for one animal. Animal IDs for open shapes: circles, Z12351; squares, Z13133; triangles, Z12216; diamonds, Z12417. Animal IDs for closed shapes: circles, A11213; squares, A11221; triangles, A11197; diamonds, A11198. Bars indicate maximum/minimum values. Exact p-values for significant (p < 0.05) and near-significant differences are indicated.

Despite our finding that ~4% ΔCCR5 peripheral blood cells are insufficient for HIV cure, this intervention may have significantly impacted the size of the latent SHIV reservoir in secondary lymphoid tissues, which likely act as key sites of virus persistence. To test this possibility, we first assessed the size of tissue reservoirs in stably suppressed animals. Using PCR-based assays with quantified numbers of cell inputs (**S2 Table**), we measured levels of SHIV DNA and RNA in tissues collected at necropsy in a subset of Group C ΔCCR5-transplanted animals that were necropsied while stably suppressed on cART (**Fig 1B**), comparing them to a cohort of 4 untransplanted controls in Group E that were infected and necropsied following stable suppression by cART (**Fig 1C**). ΔCCR5 animals exhibited a significant reduction in the levels of SHIV DNA in lymphoid tissues (multiple lymph nodes, spleen), as well as in colon, liver, and kidney, relative to suppressed, untransplanted controls (**Fig 8A**). SHIV DNA in some tissue sites, for example basal ganglia, was driven to undetectable levels following ΔCCR5 transplant. In Group E controls, SHIV RNA was most readily detected in lymphoid tissues including lymph nodes, spleen, and tonsil, but was more variable than SHIV DNA, consistent with past findings in patients and NHP models [30–32] (**Fig 8B**). ΔCCR5 animals showed significant/near significant reductions in SHIV RNA in inguinal and submandibular nodes, tonsil, and rectum, while an increase in SHIV RNA expression was observed in other tissues, such as duodenum. Next, we supplemented PCR-based tissue reservoir assays with DNAscope- and RNAscope-based *in situ* assays. Although Group E controls were not available for histological analysis, we nevertheless characterized SHIV DNA^+^ and RNA^+^ cells in animals that received ΔCCR5 HSPCs before SHIV infection (Group A), or following infection and stable suppression with (Group B) or without (Group C) subsequent cART withdrawal. Productively infected (SHIV RNA^+^) cells were detected in all tissues in Groups A-C (**S6 Fig**). Again consistent with past reports [31–33], these cells were found preferentially in B-cell follicles (BCFs) and lymphoid aggregates within secondary lymphoid tissues including lymph nodes, spleen, tonsil, thymus, and gut (**S6A–S6B Fig**). In infected animals that were transplanted and necropsied while stably suppressed (Group C), ongoing viral RNA expression (vRNA+ cells) was most readily detected in lymph nodes and GI tract tissue; however, vRNA+ cells were found in all tissue compartments including the male genital tract and CNS **S6B Fig**). Consistent with the possibility of ongoing viral replication and virion production during suppressive cART, we consistently found virions trapped on follicular dendritic cells (FDCs) within BCFs of animals that were transplanted before infection (Group A) and after infection and suppression (Group C) (**S6C Fig**). In contrast to SHIV RNA levels, which differed substantially between groups, DNAscope measurements revealed relatively comparable levels of SHIV DNA+ cells across most tissues (**S7 Fig**). In light of the significant decreases we observed in tissue SHIV DNA and RNA levels, we conclude that the impact of ΔCCR5 transplantation in infected, suppressed animals is primarily manifest in tissues.

## Discussion

Hematopoietic stem cell transplantation has led to the most dramatic HIV reservoir reductions observed in patients [1, 14, 34], yet such interventions are currently also the least practical. Here, we describe a nonhuman primate model of suppressed HIV infection that facilitates the translation of gene and cell therapy-based cure approaches to the clinic. We show that autologous, CCR5-edited HSPCs engraft in infected and uninfected animals, undergo virus-dependent positive selection, and impact viral reservoirs primarily in tissues.

We have previously utilized our model to identify immunological correlates of viral rebound following autologous transplantation with unmodified HSPCs [6]. Here, we added CCR5 gene editing, and evaluated impacts on latently infected cells. ΔCCR5 cells engraft with similar efficiency and kinetics in uninfected and infected animals, demonstrating that latent infection does not impact the ability of these cells to persist in peripheral blood and in tissues. However, our data suggest that the percentage of long-term persisting, CCR5-edited cells that are achievable with our current methods (~4% of total white blood cells) do not meet the minimum critical threshold necessary to induce viral remission in the absence of suppressive therapy. We are currently developing optimized culture conditions and maximizing the efficiency of our gene editing techniques, in order to increase the persistence of long-term engrafting, CCR5-edited HSPCs and their progeny. Importantly, even at present levels of engraftment, active SHIV replication seems to drive enrichment of ΔCCR5 CD4^+^ T-cells as they differentiate into effector memory (up to 15-fold enrichment) and terminally differentiated phenotypes (up to 56-fold enrichment). In animals that were transplanted after infection and stable suppression, rebound SHIV viremia drove up to 9- and 31-fold enrichment in these respective subsets. While we were not able to assign statistical significance to SHIV-dependent positive selection in CD4^+^ subsets, these data suggest that even at low levels, ΔCCR5 HSPCs may give rise to infection-resistant ΔCCR5 CD4^+^ T-cells that refill the SHIV-depleted CD4^+^ T-cell niche. Approaches involving the direct infusion of ΔCCR5 T-cells, have shown promise in clinical trials [35]. Importantly, a purely “defensive” strategy, such as CCR5 gene editing, may be necessary but insufficient for HIV cure. Future iterations of gene therapy-mediated cure approaches should focus on modification strategies that protect cells and augment virus-specific immunity in order to actively target reservoir sites during ongoing suppressive therapy. Chimeric antigen receptors, DARTs, and broadly neutralizing antibodies are among many approaches that could be combined with, or integrated into gene therapy-mediated HIV cure strategies [36–40].

Recent findings from multiple groups suggest that tissue reservoirs may be distinct from peripheral reservoirs due to limitations in penetration of cART compounds [41, 42], trafficking of infected cells [41, 43, 44], and other anatomical barriers [45]. We extensively examined tissue viral reservoirs by measuring tissue-associated levels of SHIV RNA and DNA using multiple assays, and compared these findings to QVOA-based measurements of the peripheral reservoir. Because myeloablative conditioning regimens such as total body irradiation (TBI) deplete peripheral CD4^+^ T-cells more efficiently than tissue-associated cells [7], we predicted that autologous transplantation would have a greater impact on the peripheral reservoir relative to tissue reservoirs. On the contrary, we demonstrate that autologous transplantation primarily impacts latently infected cells in tissue reservoirs rather than peripheral blood reservoirs. Tissue-associated SHIV DNA and RNA levels in suppressed, transplanted animals were significantly lower than those in suppressed, untransplanted controls, especially in tissues that are known to harbor replication competent virus during suppressive therapy. In contrast, the size of the peripheral reservoir, measured by QVOA, was not significantly different in transplanted vs. untransplanted animals. We conclude that transplantation primarily impacts tissue reservoirs, whereas effects in the peripheral reservoir are secondary.

Our study was unable to directly address whether reductions in tissue-associated SHIV reservoirs were due to the transplantation regimen itself (i.e. myeloablative TBI) vs. low levels of ΔCCR5 cells. Consistent with past reports, we observed ongoing tissue-associated SHIV RNA expression in suppressed animals [30–32], as well as in suppressed, transplanted animals. PCR-based assays showed that viral RNA expression in suppressed, ΔCCR5-transplanted animals was significantly lower than untransplanted controls in multiple lymphoid tissues including lymph nodes and tonsil. However, samples from suppressed, wtCCR5-transplanted control animals, which would distinguish whether this reduction was ΔCCR5-dependent, were unavailable. Nevertheless, our viral rebound data (**Fig 4C–4E**) are consistent with a model in which increased viral replication due to myeloablative TBI [6] was offset by even low levels of ΔCCR5 HSPCs and their progeny. These results are highly promising for future approaches that combine increased levels of CCR5 editing with more active means of reservoir targeting.

Two animals in our study highlight the potential of our approach. Animals Z12420 and R10159 demonstrated a peripheral reservoir size of 0.600 IUPM and 0.064 IUPM, respectively, as measured by QVOA. Following transplantation, each was reduced to undetectable levels. Although SHIV rebound was observed in both animals following cART withdrawal, neither established a consistent plasma viral load set point. The kinetics of rebound viremia in these animals are reminiscent of “predator-prey” relationships that have been characterized between virus-specific CD8^+^ T-cells and viral escape mutants [46, 47]. This oscillatory pattern has also been correlated with T cell activation in a cohort of cART-treated patients with multi-drug resistant HIV [48], which is consistent with our observations in SHIV^+^ animals during post-transplant immune recovery [6]. The inability of a subset of ΔCCR5 animals to reestablish a consistent rebound viral set point reinforces the notion that increased efficiency gene editing approaches, combined with targeting persistently infected cells for destruction (e.g. augmenting the endogenous virus-specific immune response) represents an achievable path to HIV cure.

In conclusion, we demonstrate that ΔCCR5 HSPC gene therapy is safe and feasible in a nonhuman primate model of suppressed HIV infection. ΔCCR5 HSPCs persist long term, and HSPC-derived ΔCCR5 CD4^+^ T-cells expand during active SHIV replication. We observe a primary and significant impact of this therapy on tissue reservoirs. Increased efficiency CCR5-editing strategies could further decrease the number of latently infected cells in these compartments, and would be significantly augmented by strategies designed to actively target latently infected cells and/or enhance the host response to recrudescent virus. Our model is ideally suited both to characterize key sites of HIV persistence, and target them with combination therapies.

## Supporting information

S1 Table Summary of study animals. (DOCX)

S2 Table Input genomes for SHIV DNA and RNA measurements. (XLSX)

S1 Fig Plasma viral loads in Group C animals. At the indicated weeks following IV infection with SHIV-C, PCR-based methods were used to measure plasma viral load (PVL) in the indicated animal from study group C. Arrow indicates initiation of cART, which was maintained through necropsy (dagger). Animals underwent autologous hematopoietic stem cell transplant between weeks 53 and 60.
(DOCX)

S2 Fig Plasma viral loads in Group E animals. At the indicated weeks following IV infection with SHIV-C, PCR-based methods were used to measure plasma viral load (PVL) in the indicated animals from study group E. Arrow indicates initiation of cART, which was maintained through necropsy (dagger).
(DOCX)

S3 Fig Longitudinal tissue viral loads in animals transplanted prior to SHIV challenge. Group A animals (n = 4) were transplanted with ΔCCR5 HSPCs approximately 6 months prior to IV challenge with SHIV-C. At the indicated weeks post SHIV challenge, duodenal/jejunual biopsies (“Upper GI,” [panels **A** and **B**]), colonic biopsies (“Lower GI,” [panels **C** and **D**]), and peripheral lymph nodes (Axillary/Inguinal, [panels **E** and **F**]) were collected. SHIV DNA (panels **A, C, D**) or SHIV RNA (panels **B, D, F**) were measured by real-time PCR. Controls represent available time point-matched samples from 16-24 untransplanted, infected animals derived from Groups B-E.
(DOCX)

S4 Fig CD4^+^ T-cell subset percentages in transplanted animals before and after SHIV challenge. Upper (duodenum/jejunum; [panels **A, C, E**]) and lower GI biopsies (colon; [panels **B, D, F**]) were collected from Group A animals that received CCR5-edited HSPCs prior to SHIV infection (“ΔCCR5 Transplant,” open circles), and compared to control animals (closed circles) derived from Groups D-E that were not transplanted prior to infection. Shown are total CD3^+^CD4^+^ cells (panels **A-B**), Central Memory CD4^+^ T-cells (T_CM_, panels **C-D**), and Effector memory CD4^+^ T-cells (T_EM_, panels **E-F**) measured by flow cytometry from enzymatically dissociated specimens. Memory subsets were distinguished on the basis of CD45RA and CCR7 expression (see materials and methods). Upper GI sampling was only conducted in animals larger than 3kg.
(DOCX)

S5 Fig Longitudinal tissue viral loads in animals transplanted during suppressed SHIV infection. Group B-C animals (n = 13) were transplanted with ΔCCR5 HSPCs approximately 12 months after IV challenge with SHIV-C, and 6 months after initiation of cART. At the indicated weeks post cART initiation, duodenal/jejunual biopsies (“Upper GI,” [panels **A** and **B**]), colonic biopsies (“Lower GI,” [panels **C** and **D**]), and peripheral lymph nodes (Axillary/Inguinal, [panels **E** and **F**]) were collected. SHIV DNA (panels **A, C, E**) or SHIV RNA (panels **B, D, F**) were measured by real-time PCR. Exact p-values are indicated.
(DOCX)

S6 Fig RNAscope analyses of SHIV tissue RNA. Animals from Groups A (n = 4), B (n = 5) and C (n = 6) were transplanted with ΔCCR5 HSPCs as described in Fig 1, and tissue sections were prepared at necropsy for SHIV RNAscope analysis. **(A)**: SHIV RNA^+^ cells/10^6^ cells from Group A. **(B)**: SHIV RNA^+^ cells/10^6^ cells from Groups B-C. **(C)** SHIV Virions/10^6^ cells from B-Cell Follicles (“BCF”) or Lymphoid Aggregates (“LAgg”) from Groups A-C. TCZ: T-Cell Zone; WP: White Pulp; LP: Lamina Propria; LN: Lymph Node.
(DOCX)

S7 Fig DNAscope analyses of SHIV tissue DNA. Animals from Groups A (n = 4), B (n = 6) and C (n = 6) were transplanted with ΔCCR5 HSPCs as described in Fig 1, and tissue sections were prepared at necropsy for SHIV DNAscope analysis. Shown are SHIV DNA^+^ cells/10^6^ cells from Group A **(A)**, Groups B-C **(B)**, and B-Cell Follicles (“BCF”) or Lymphoid Aggregates (“LAgg”) from Groups A-C **(C)**. TCZ: T-Cell Zone; WP: White Pulp; LP: Lamina Propria; LN: Lymph Node.
(DOCX)
