# Supplementary material for: Differential impact of transplantation on peripheral and tissue-associated viral reservoirs: Implications for HIV gene therapy
Source: PLoS Pathog. 2018 Apr 19;14(4):e1006956. doi: 10.1371/journal.ppat.1006956 (PMC5908070; doi:10.1371/journal.ppat.1006956)
Supplement: S1 Table — (DOCX) [file ppat.1006956.s001.docx]

**S1 Table.** Summary of study animals.

| Group | Animal IDs | Notes |
| --- | --- | --- |
| Group A: ΔCCR5 Transplant-SHIV | M10127 | Infusion Data Only |
|  | A11217 | [23] |
|  | A11210 | [23] |
|  | Z12161 | [23] |
|  | Z12220 | [23] |
| Group B: SHIV-cART- ΔCCR5 Transplant | A11219 | Necropsied following cART Withdrawal |
|  | T10187 | Necropsied following cART Withdrawal |
|  | R10159 | Necropsied following cART Withdrawal |
|  | T10173 | Necropsied following cART Withdrawal |
|  | Z11151 | Necropsied following cART Withdrawal |
|  | Z12420 | Necropsied following cART Withdrawal |
| Group C: SHIV-cART- ΔCCR5 Transplant | R10155 | Necropsied on cART |
|  | Z12216 | Necropsied on cART |
|  | Z12037 | Necropsied on cART |
|  | Z12351 | Necropsied on cART |
|  | Z12370 | Necropsied on cART |
|  | Z13133 | Necropsied on cART |
|  | Z12417 | Necropsied on cART |
| Group D: SHIV-cART-No Transplant | Z09087 | [6] |
|  | Z09106 | [6] |
|  | Z09192 | [6] |
|  | Z09204 | [6] |
|  | A11201 | [6] |
| Group E: SHIV-cART | A11197 |  |
|  | A11198 |  |
|  | A11213 |  |
|  | A11221 |  |
| Group F: SHIV-cART-wt CCR5 Transplant | Z09144 | [6] |
|  | Z08214 | [6] |
|  | A11200 | [6] |
|  | Z09196 | [6] |
|  | Z09125 | [6] |
