# Supplementary material for: Differential impact of transplantation on peripheral and tissue-associated viral reservoirs: Implications for HIV gene therapy
Source: PLoS Pathog. 2018 Apr 19;14(4):e1006956. doi: 10.1371/journal.ppat.1006956 (PMC5908070; doi:10.1371/journal.ppat.1006956)
Supplement: S1 Fig — At the indicated weeks following IV infection with SHIV-C, PCR-based methods were used to measure plasma viral load (PVL) in the indicated animal from study group C. Arrow indicates initiation of cART, which was maintained through necropsy (dagger). Animals underwent autologous hematopoietic stem cell transplant between weeks 53 and 60. (DOCX) [file ppat.1006956.s003.docx]

**
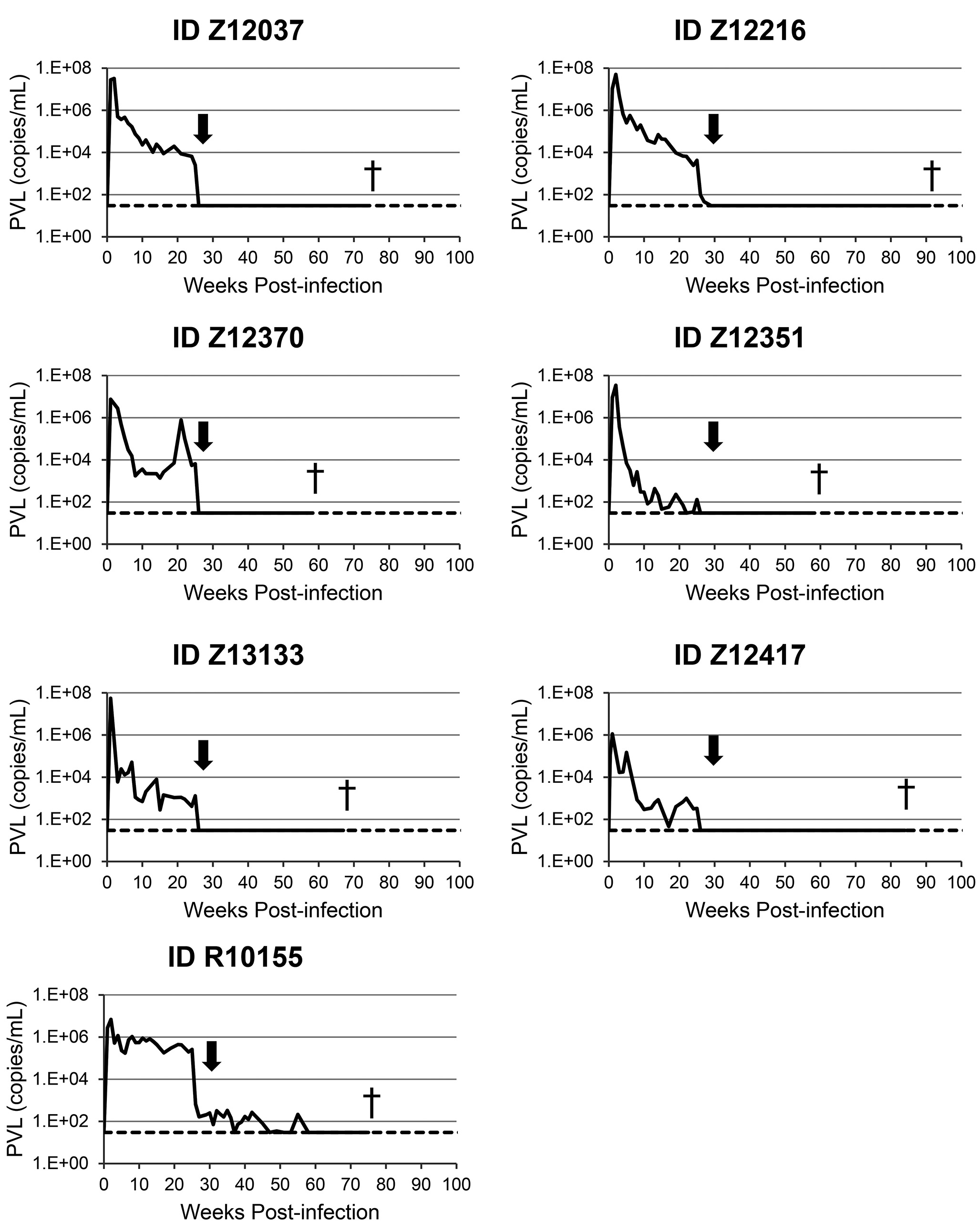
**

**S1 Fig. Plasma viral loads in Group C animals.** At the indicated weeks following IV infection with SHIV-C, PCR-based methods were used to measure plasma viral load (PVL) in the indicated animal from study group C. Arrow indicates initiation of cART, which was maintained through necropsy (dagger). Animals underwent autologous hematopoietic stem cell transplant between weeks 53 and 60.
