# Supplementary material for: Differential impact of transplantation on peripheral and tissue-associated viral reservoirs: Implications for HIV gene therapy
Source: PLoS Pathog. 2018 Apr 19;14(4):e1006956. doi: 10.1371/journal.ppat.1006956 (PMC5908070; doi:10.1371/journal.ppat.1006956)
Supplement: S2 Fig — At the indicated weeks following IV infection with SHIV-C, PCR-based methods were used to measure plasma viral load (PVL) in the indicated animals from study group E. Arrow indicates initiation of cART, which was maintained through necropsy (dagger). (DOCX) [file ppat.1006956.s004.docx]

**
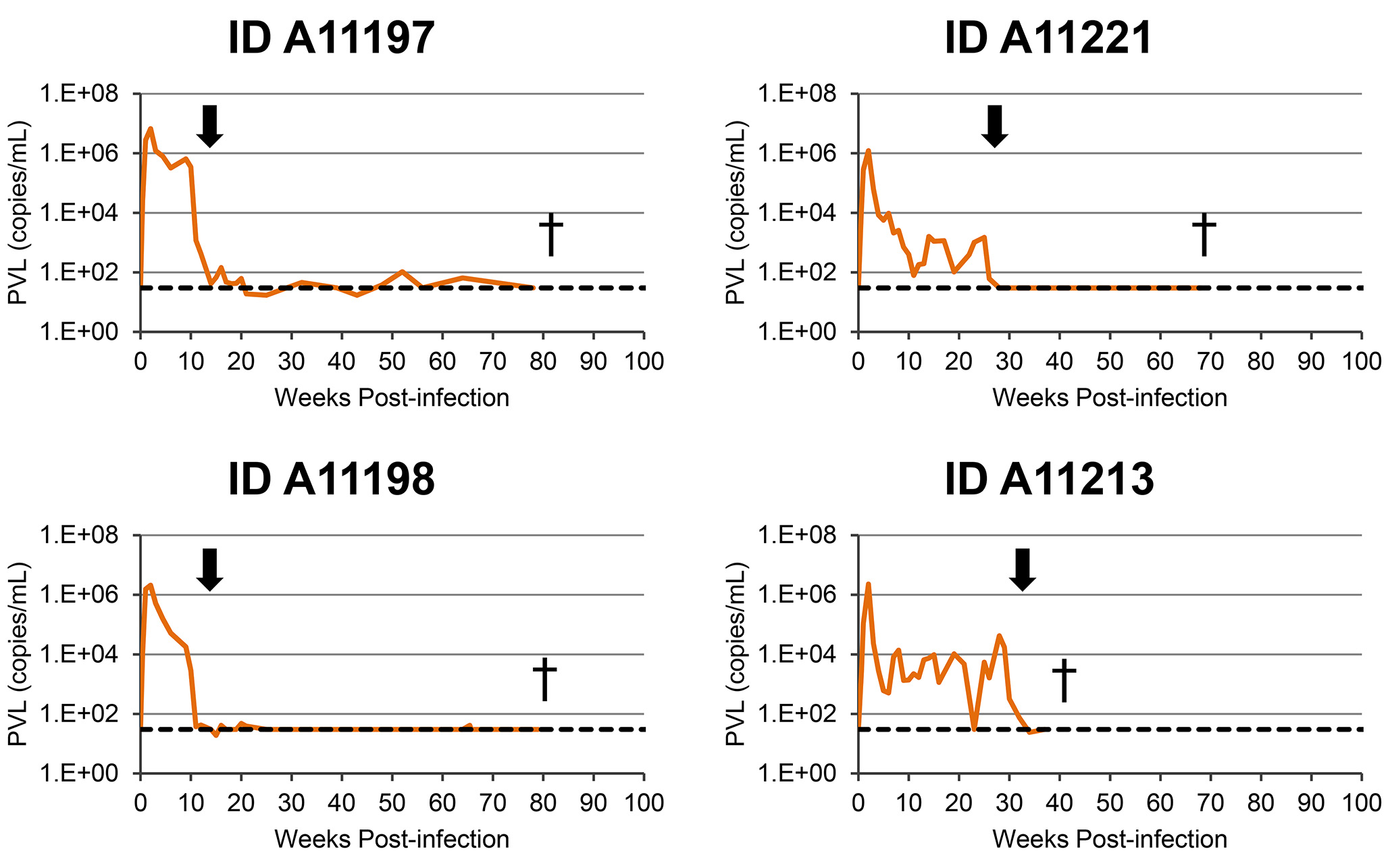
**

**S2 Fig. Plasma viral loads in Group E animals.** At the indicated weeks following IV infection with SHIV-C, PCR-based methods were used to measure plasma viral load (PVL) in the indicated animals from study group E. Arrow indicates initiation of cART, which was maintained through necropsy (dagger).
