# Supplementary material for: Differential impact of transplantation on peripheral and tissue-associated viral reservoirs: Implications for HIV gene therapy
Source: PLoS Pathog. 2018 Apr 19;14(4):e1006956. doi: 10.1371/journal.ppat.1006956 (PMC5908070; doi:10.1371/journal.ppat.1006956)
Supplement: S3 Fig — Group A animals (n = 4) were transplanted with ΔCCR5 HSPCs approximately 6 months prior to IV challenge with SHIV-C. At the indicated weeks post SHIV challenge, duodenal/jejunual biopsies (“Upper GI,” [panels A and B]), colonic biopsies (“Lower GI,” [panels C and D]), and peripheral lymph nodes (Axillary/Inguinal, [panels E and F]) were collected. SHIV DNA (panels A, C, D) or SHIV RNA (panels B, D, F) were measured by real-time PCR. Controls represent available time point-matched samples from 16-24 untransplanted, infected animals derived from Groups B-E. (DOCX) [file ppat.1006956.s005.docx]

**
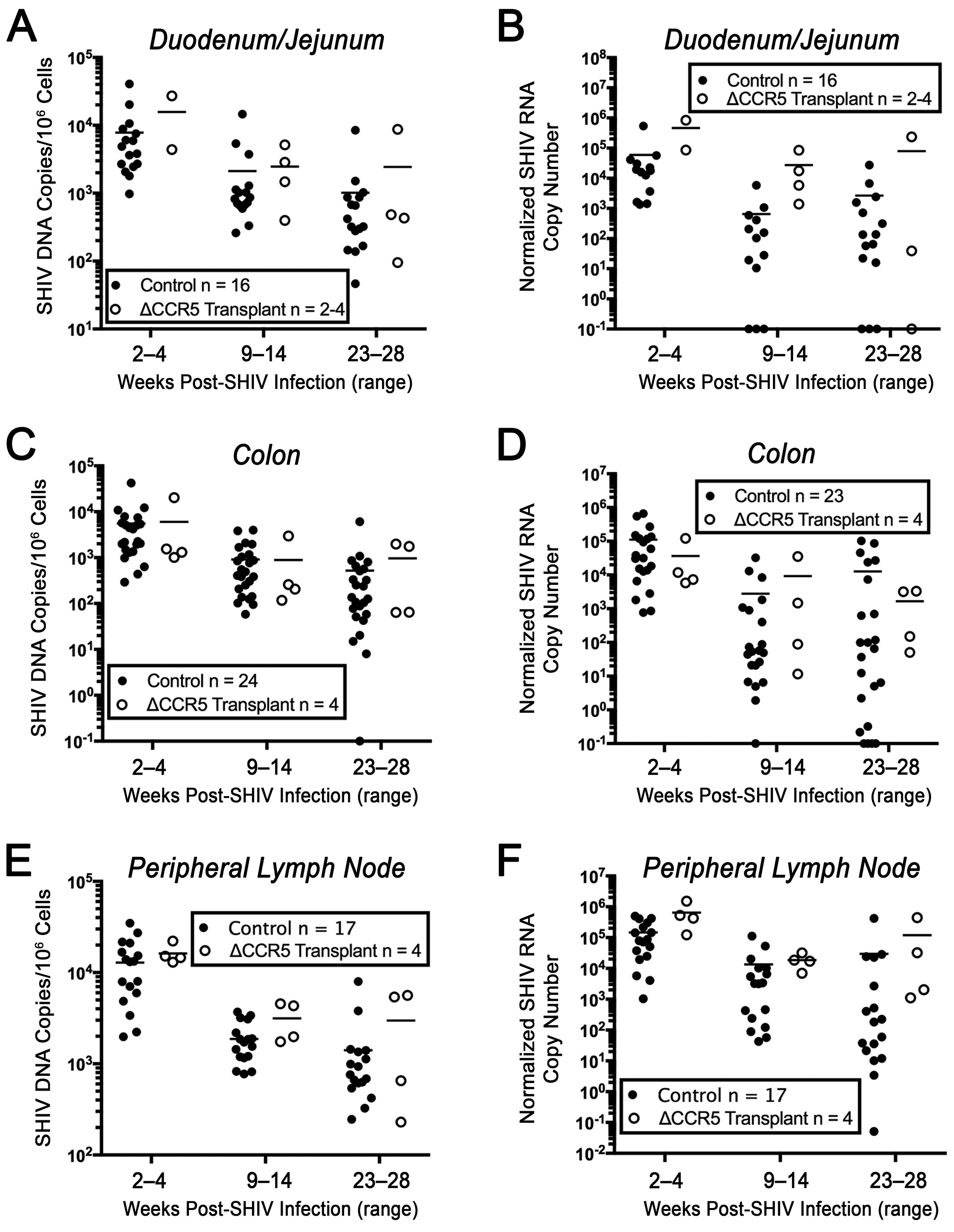
**

**S3 Fig. Longitudinal tissue viral loads in animals transplanted prior to SHIV challenge.** Group A animals (n=4) were transplanted with ΔCCR5 HSPCs approximately 6 months prior to IV challenge with SHIV-C. At the indicated weeks post SHIV challenge, duodenal/jejunual biopsies (“Upper GI,” [panels **A** and **B**]), colonic biopsies (“Lower GI,” [panels **C** and **D**]), and peripheral lymph nodes (Axillary/Inguinal, [panels **E** and **F**]) were collected. SHIV DNA (panels **A,C, D**) or SHIV RNA (panels **B,D, F**) were measured by real-time PCR. Controls represent available time point-matched samples from 16-24 untransplanted, infected animals derived from Groups B-E.
