# Supplementary material for: Differential impact of transplantation on peripheral and tissue-associated viral reservoirs: Implications for HIV gene therapy
Source: PLoS Pathog. 2018 Apr 19;14(4):e1006956. doi: 10.1371/journal.ppat.1006956 (PMC5908070; doi:10.1371/journal.ppat.1006956)
Supplement: S4 Fig — Upper (duodenum/jejunum; [panels A, C, E]) and lower GI biopsies (colon; [panels B, D, F]) were collected from Group A animals that received CCR5-edited HSPCs prior to SHIV infection (“ΔCCR5 Transplant,” open circles), and compared to control animals (closed circles) derived from Groups D-E that were not transplanted prior to infection. Shown are total CD3+CD4+ cells (panels A-B), Central Memory CD4+ T-cells (TCM, panels C-D), and Effector memory CD4+ T-cells (TEM, panels E-F) measured by flow cytometry from enzymatically dissociated specimens. Memory subsets were distinguished on the basis of CD45RA and CCR7 expression (see materials and methods). Upper GI sampling was only conducted in animals larger than 3kg. (DOCX) [file ppat.1006956.s006.docx]

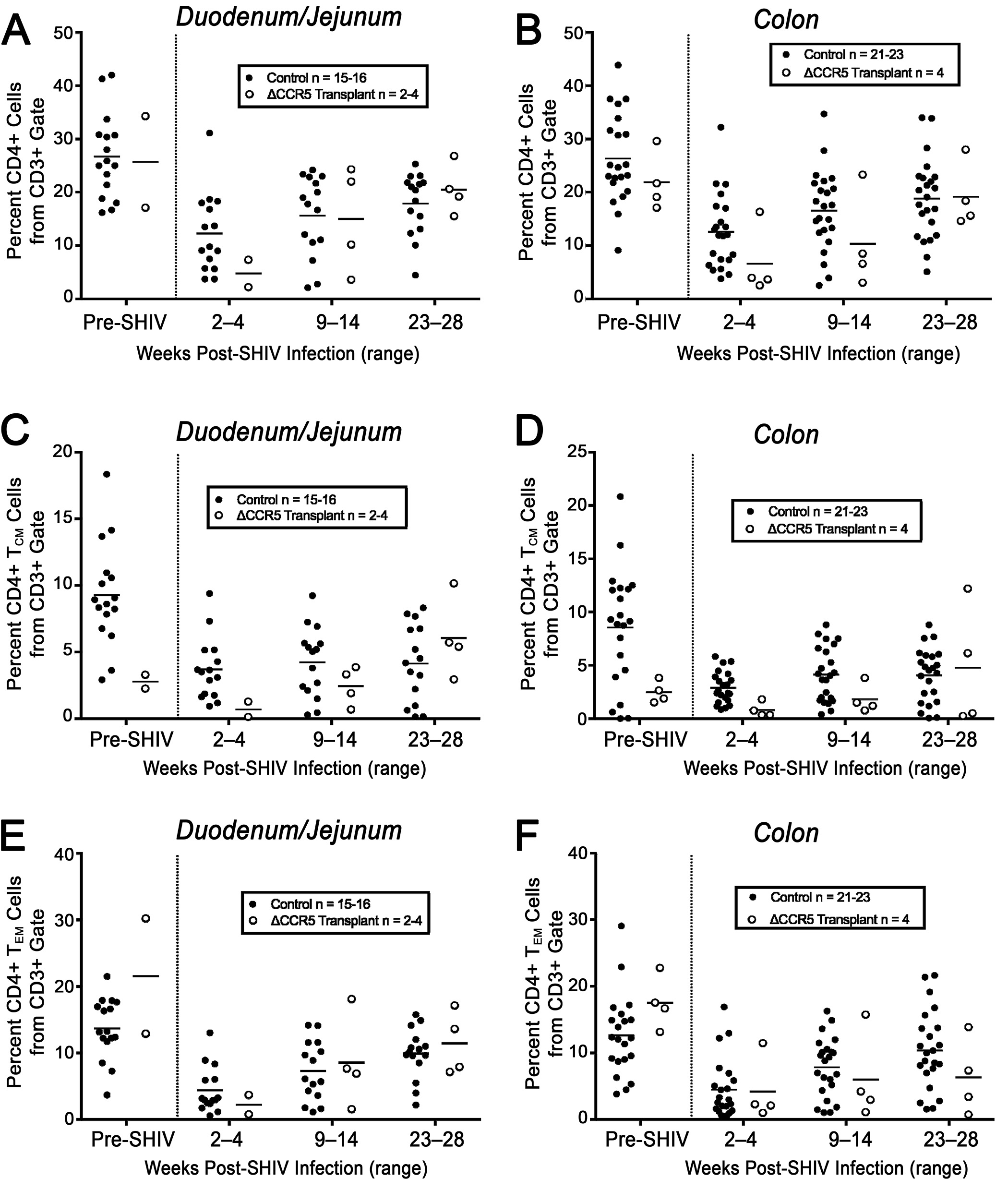


**S4 Fig. CD4^+^ T-cell subset percentages in transplanted animals before and after SHIV challenge.** Upper (duodenum/jejunum; [panels **A, C, E**]) and lower GI biopsies (colon; [panels **B, D, F**]) were collected from Group A animals that received CCR5-edited HSPCs prior to SHIV infection (“ΔCCR5 Transplant,” open circles), and compared to control animals (closed circles) derived from Groups D-E that were not transplanted prior to infection. Shown are total CD3^+^CD4^+^ cells (panels **A-B**), Central Memory CD4^+^ T-cells (T_CM_, panels **C-D**), and Effector memory CD4^+^ T-cells (T_EM_, panels **E-F**) measured by flow cytometry from enzymatically dissociated specimens. Memory subsets were distinguished on the basis of CD45RA and CCR7 expression (see materials and methods). Upper GI sampling was only conducted in animals larger than 3kg.
