# Supplementary material for: Differential impact of transplantation on peripheral and tissue-associated viral reservoirs: Implications for HIV gene therapy
Source: PLoS Pathog. 2018 Apr 19;14(4):e1006956. doi: 10.1371/journal.ppat.1006956 (PMC5908070; doi:10.1371/journal.ppat.1006956)
Supplement: S5 Fig — Group B-C animals (n = 13) were transplanted with ΔCCR5 HSPCs approximately 12 months after IV challenge with SHIV-C, and 6 months after initiation of cART. At the indicated weeks post cART initiation, duodenal/jejunual biopsies (“Upper GI,” [panels A and B]), colonic biopsies (“Lower GI,” [panels C and D]), and peripheral lymph nodes (Axillary/Inguinal, [panels E and F]) were collected. SHIV DNA (panels A, C, E) or SHIV RNA (panels B, D, F) were measured by real-time PCR. Exact p-values are indicated. (DOCX) [file ppat.1006956.s007.docx]

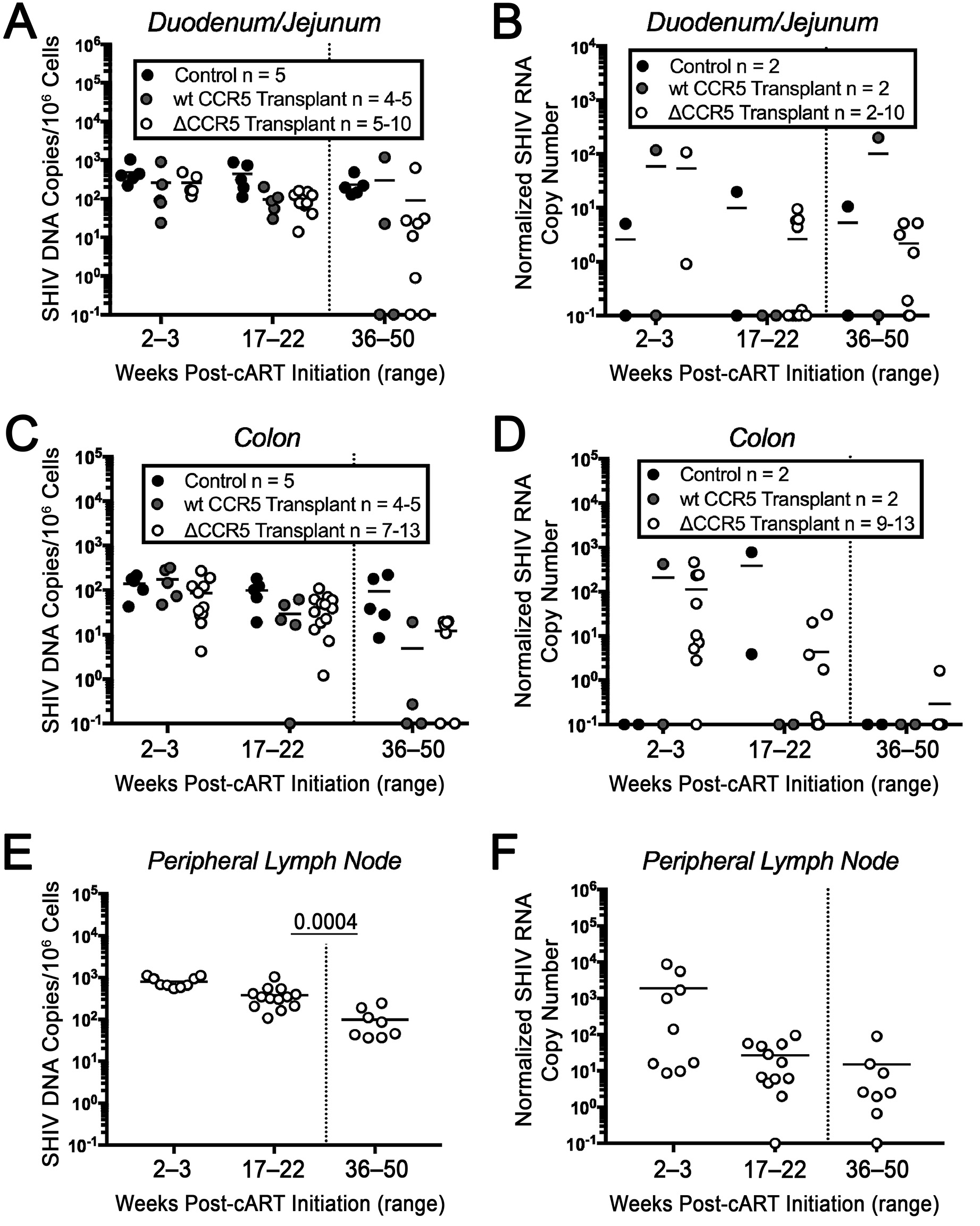


**S5 Fig. Longitudinal tissue viral loads in animals transplanted during suppressed SHIV infection.** Group B-C animals (n= 13) were transplanted with ΔCCR5 HSPCs approximately 12 months after IV challenge with SHIV-C, and 6 months after initiation of cART. At the indicated weeks post cART initiation, duodenal/jejunual biopsies (“Upper GI,” [panels **A** and **B**]), colonic biopsies (“Lower GI,” [panels **C** and **D**]), and peripheral lymph nodes (Axillary/Inguinal, [panels **E** and **F**]) were collected. SHIV DNA (panels **A, C, E**) or SHIV RNA (panels **B, D, F**) were measured by real-time PCR. Exact p-values are indicated.
