# Supplementary material for: Differential impact of transplantation on peripheral and tissue-associated viral reservoirs: Implications for HIV gene therapy
Source: PLoS Pathog. 2018 Apr 19;14(4):e1006956. doi: 10.1371/journal.ppat.1006956 (PMC5908070; doi:10.1371/journal.ppat.1006956)
Supplement: S6 Fig — Animals from Groups A (n = 4), B (n = 5) and C (n = 6) were transplanted with ΔCCR5 HSPCs as described in Fig 1, and tissue sections were prepared at necropsy for SHIV RNAscope analysis. (A): SHIV RNA+ cells/106 cells from Group A. (B): SHIV RNA+ cells/106 cells from Groups B-C. (C) SHIV Virions/106 cells from B-Cell Follicles (“BCF”) or Lymphoid Aggregates (“LAgg”) from Groups A-C. TCZ: T-Cell Zone; WP: White Pulp; LP: Lamina Propria; LN: Lymph Node. (DOCX) [file ppat.1006956.s008.docx]

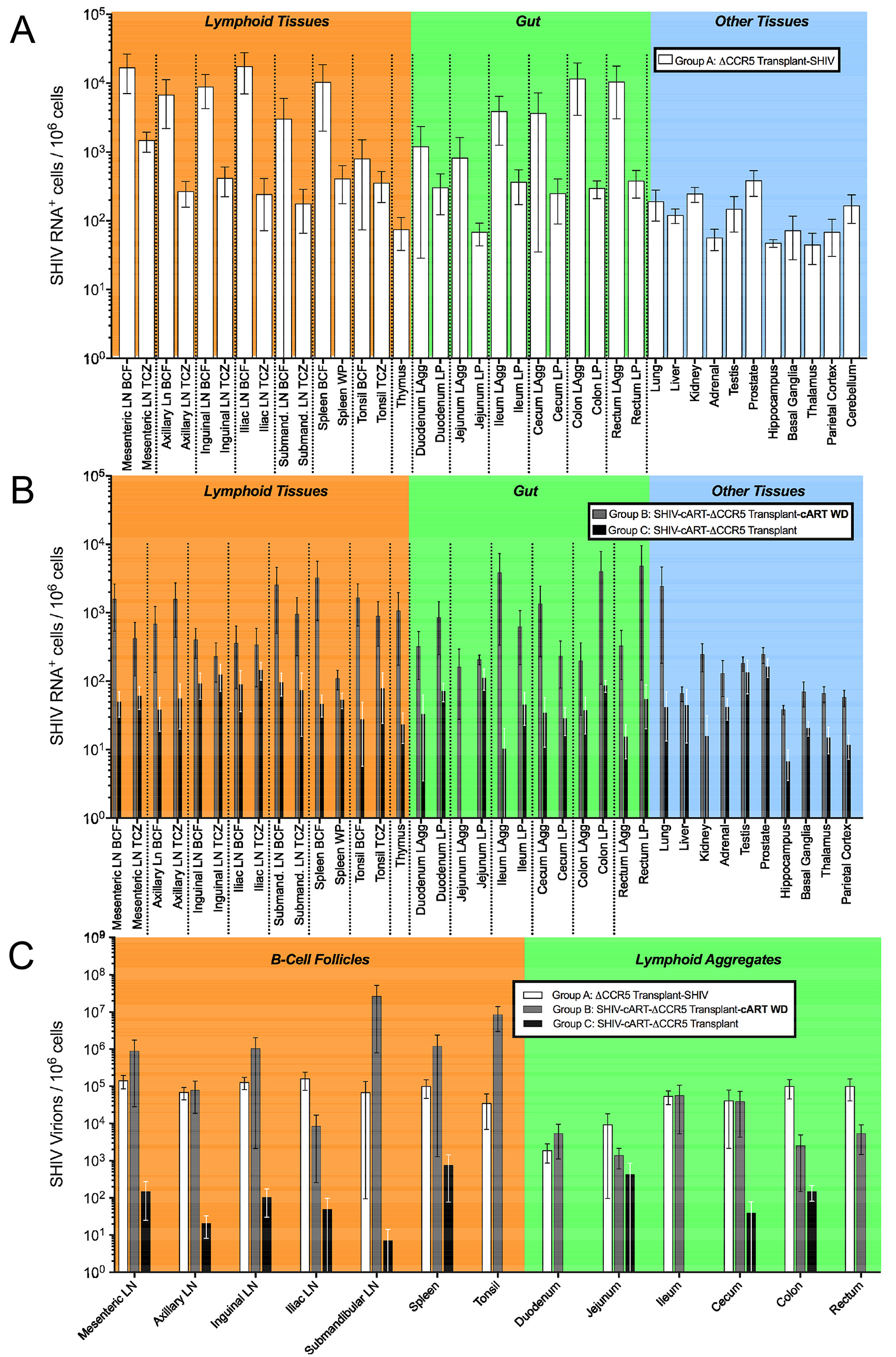


**S6 Fig. RNAscope analyses of SHIV tissue RNA.** Animals from Groups A (n = 4), B (n = 5) and C (n = 6) were transplanted with ΔCCR5 HSPCs as described in Figure 1, and tissue sections were prepared at necropsy for SHIV RNAscope analysis. **(A)**: SHIV RNA^+^ cells/10^6^ cells from Group A. **(B)**: SHIV RNA^+^ cells/10^6^ cells from Groups B-C. **(C)** SHIV Virions/10^6^ cells from B-Cell Follicles (“BCF”) or Lymphoid Aggregates (“LAgg”) from Groups A-C. TCZ: T-Cell Zone; WP: White Pulp; LP: Lamina Propria; LN: Lymph Node.
