# Supplementary material for: Differential impact of transplantation on peripheral and tissue-associated viral reservoirs: Implications for HIV gene therapy
Source: PLoS Pathog. 2018 Apr 19;14(4):e1006956. doi: 10.1371/journal.ppat.1006956 (PMC5908070; doi:10.1371/journal.ppat.1006956)
Supplement: S7 Fig — Animals from Groups A (n = 4), B (n = 6) and C (n = 6) were transplanted with ΔCCR5 HSPCs as described in Fig 1, and tissue sections were prepared at necropsy for SHIV DNAscope analysis. Shown are SHIV DNA+ cells/106 cells from Group A (A), Groups B-C (B), and B-Cell Follicles (“BCF”) or Lymphoid Aggregates (“LAgg”) from Groups A-C (C). TCZ: T-Cell Zone; WP: White Pulp; LP: Lamina Propria; LN: Lymph Node. (DOCX) [file ppat.1006956.s009.docx]

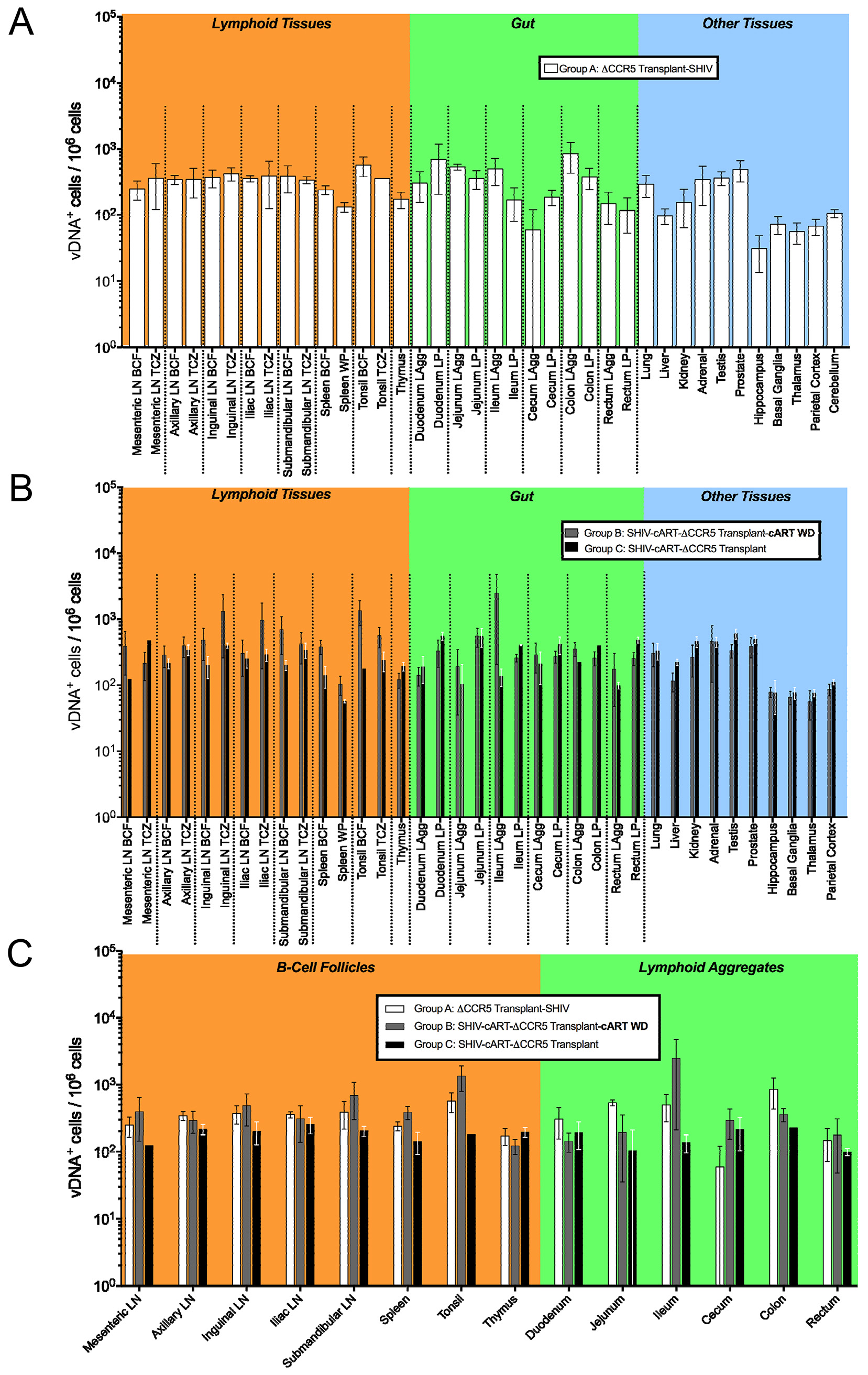


**S7 Fig. DNAscope analyses of SHIV tissue DNA.** Animals from Groups A (n = 4), B (n = 6) and C (n = 6) were transplanted with ΔCCR5 HSPCs as described in Figure 1, and tissue sections were prepared at necropsy for SHIV DNAscope analysis. Shown are SHIV DNA^+^ cells/10^6^ cells from Group A **(A)**, Groups B-C **(B)**, and B-Cell Follicles (“BCF”) or Lymphoid Aggregates (“LAgg”) from Groups A-C **(C)**. TCZ: T-Cell Zone; WP: White Pulp; LP: Lamina Propria; LN: Lymph Node.
